# Deployability of machine-learning prediction models in elite and high-level team sports: a task-specific systematic review

**DOI:** 10.3389/fphys.2026.1920214

**Published:** 2026-09-30

**Authors:** Zhiyu Guan, Zicheng Jin, Chuangui Mao, Weiguo Liu, Jie Ren

**Affiliations:** 1 College of Physical Education and Health, Guangxi Normal University, Guilin, China; 2 Department of Physical Education, Guilin Medical University, Guilin, China

**Keywords:** data leakage, deployability, machine learning, prediction models, PROBAST, team sports

## Abstract

Machine-learning models are increasingly used for match forecasting and athlete monitoring in elite and high-level team sports, but high reported performance does not necessarily indicate readiness for real-world use. This systematic review evaluated whether models were developed and tested under conditions compatible with their intended decision time. Eight databases were searched, with searches completed by 12 July 2026. Eighty reports representing 77 studies contributed 87 analysis units: 55 Core, 23 post-event or high-leakage Comparators, and nine Boundary units. All 87 units were assessed for deployable prediction validity (DPV) across six domains, and 78 Core or Comparator units were also assessed with PROBAST. Amongst Core units, 26/55 (47.3%) were rated at least Moderate, including five Moderate-to-strong and 21 Moderate units. Pre-adjudication agreement for the overall DPV classification was 85/87 (97.7%; κ = 0.968; linearly weighted κ = 0.978), whilst domain-level exact agreement ranged from 75/87 to 87/87. In *post hoc* sensitivity analyses, 19/55 Core units met the primary DPV threshold and had Sufficient validation; 14/55 met the primary threshold and had at least partial evidence of predictive reliability or probability-quality; and 9/55 met the primary threshold and both additional conditions. PROBAST risk of bias was High for 56/78 units, Unclear for 21/78, and Low for one. The main limitations were evaluation designs that did not adequately reflect intended future use and limited assessment of predictive reliability. Overall, the evidence supports selective methodological readiness rather than general deployability, with different requirements for pre-game, in-game, and athlete-monitoring applications.

**Systematic review registration:**

https://www.crd.york.ac.uk/PROSPERO/view/CRD420261425179 **, identifier CRD420261425179.**

## 1 Introduction

Elite and high-level team-sport organisations routinely collect match statistics, rankings, tracking data, wearable-sensor outputs, training-load measures, ratings of perceived exertion, wellness reports, and physiological signals to guide performance practice (Akenhead and Nassis, 2016; Bourdon et al., 2017; Camomilla et al., 2018; Halson, 2014; Impellizzeri et al., 2019). Machine-learning models use these data for match forecasting, athlete monitoring, internal-load estimation, recovery assessment, and longer-term performance questions. For these models to inform practice, their outputs need to be available when a decision is made and evaluated in data that reflect the athletes, matches, or periods in which they are expected to be used.

Previous reviews have described a wide range of artificial-intelligence and machine-learning applications in team sports. Claudino et al. reviewed injury-risk assessment and performance prediction, Bunker and Susnjak examined match-result prediction, Tang et al. focused on talent identification, and Dehbane et al. reviewed technologies for performance enhancement (Claudino et al., 2019; Bunker and Susnjak, 2022; Dehbane et al., 2026; Tang et al., 2026). Other reviews have addressed big-data and tactical analytics and machine learning for sport-injury prediction (Rein and Memmert, 2016; Goes et al., 2021; Van Eetvelde et al., 2021). Together, this literature shows broad interest in machine learning, whilst repeatedly identifying small samples, limited independent validation, inconsistent reporting, and data leakage as common concerns. The practical consequences of these problems depend on the specific decision a model is meant to support.

The timing of information can change what a prediction means in practice. Complete-match box scores are available only after a match has finished, and measurements from a training session become available as that session progresses. These data can support retrospective analysis or later monitoring decisions, but they cannot inform an earlier decision. Reported performance can also be optimistic when related observations are split across development and evaluation data or when evaluation data influence preprocessing or model selection (Varma and Simon, 2006; Kaufman et al., 2012). Model explainability methods such as SHAP do not remove these design concerns.

Evaluation should therefore reflect the way a model is expected to be used. Pre-game forecasts can be tested on later fixtures using predictors fixed before competition. In-game models generate repeated predictions as match information changes, so evaluation needs to account for dependence within matches. Athlete-monitoring models may forecast a future session, update an ongoing physiological response, or estimate a response after the session has been completed. Testing later sessions in the same athlete and testing entirely new athletes address different forms of generalisation. These differences also make it inappropriate to pool accuracy, area under the curve, F1 score, and prediction-error metrics across unlike tasks.

Existing prediction-model frameworks address many parts of this problem. CHARMS supports structured data extraction for prediction-model reviews, TRIPOD+AI promotes transparent reporting, and PROBAST and PROBAST+AI assess risk of bias and applicability in relation to the review question or intended use (Moons et al., 2014; Wolff et al., 2019; Collins et al., 2024; Moons et al., 2025). PROBAST+AI further makes timing, setting, and predictor assessment explicit when defining applicability to an intended use. In this review, deployable prediction validity (DPV) organised these established principles around a defined sport decision: when the model output becomes available, which inputs exist at that time, and what type of future use the evaluation can support. This perspective is especially relevant in exercise physiology, where predicting tomorrow’s wellness and estimating heart-rate response from a completed training exposure are different tasks with different decision windows.

This systematic review examined the deployability of machine-learning prediction models in elite and high-level team sports. We aimed to: (1) classify models by prediction task, intended decision time, and evidence layer; (2) assess risk of bias and applicability using PROBAST; (3) evaluate prediction timing, predictor availability, leakage control, validation strategy, calibration or task-appropriate reliability, and deployment boundaries at the analysis-unit level; and (4) identify task-specific methodological priorities for match prediction, in-game decision support, athlete monitoring, and related performance applications. The synthesis was organised around the evidence supporting each intended use.

## 2 Materials and methods

### 2.1 Review design, objective, registration, and amendments

This systematic review followed the PRISMA 2020 statement (Page et al., 2021). The PROSPERO record (CRD420261425179) was first published on 16 June 2026 and updated on 28 July 2026 to document supplemental database searches and clarify the report–study–analysis-unit structure, task- and deployment-context-based synthesis, evidence layers, and operational DPV coding; the central review question and principal DPV domains were unchanged. No separate full protocol was prepared. The review focused on whether reported model performance was obtained using information available at the intended decision time and evaluated under conditions relevant to future athletes, teams, matches, seasons, or monitoring periods. Reporting of the prediction-model synthesis was also informed by TRIPOD-SRMA (Snell et al., 2023).

We assessed six aspects of deployable prediction validity: prediction timing, predictor availability, leakage control, validation strategy, calibration or task-appropriate reliability, and deployment boundary. Reports, underlying studies, and analysis units were distinguished throughout the review, and evidence was grouped into Core, Comparator, and Boundary layers. PROBAST risk of bias and applicability were assessed separately. Full database-specific search strategies are provided in **Supplementary Table S1**.

### 2.2 Eligibility criteria

Eligible populations included athletes, teams, or competitions from elite or high-level team-sport settings, such as professional, semi-professional, national-level, and top-tier collegiate competition. Semi-professional and national-level samples were included when the setting clearly represented high-performance sport. Recreational samples were excluded.

Eligible reports evaluated at least one machine-learning, deep-learning, artificial-intelligence, or ensemble prediction model. Prediction targets included pre-game match outcomes or scores; in-game probabilities or future match states; training load, perceived exertion, wellness, fatigue, recovery, readiness, physiological response, or adaptation; season-level performance, playing time, availability, or longer-term player outcomes; and related performance decisions. We included retrospective and prospective observational studies, longitudinal monitoring studies, database studies, model-development studies, validation studies, and model-comparison studies when the report contained enough information to assess the prediction target, predictor timing, validation strategy, and model performance.

We excluded reviews, commentaries, editorials, non-team-sport studies, recreational samples, studies without an eligible machine-learning prediction model, pure action-recognition or detection tasks, and reports with insufficient model information. Studies using complete target-event data, post-event statistics, or future information were retained as post-event or high-leakage Comparators when they offered a useful methodological contrast. They were not used to support conclusions about pre-decision deployment. Related applications outside the main prediction and monitoring tasks were retained as Boundary evidence and interpreted separately.

### 2.3 Information sources and search strategy

The original searches covered PubMed, Embase, Web of Science, and the Cochrane Library from database inception to 6 May 2026. Search results were exported from each database and imported into EndNote X9 (Clarivate Analytics) for reference management. The imported records comprised 5,261 from PubMed, 1,033 from Embase, 763 from Web of Science, and 93 from the Cochrane Library, yielding 7,150 records before duplicate removal.

Supplemental searches were conducted on 12 July 2026 in Scopus, SPORTDiscus via EBSCOhost, IEEE Xplore, and the ACM Digital Library. Records from these searches were imported into the same EndNote X9 library. The searches identified 2,116 records from Scopus, 475 from SPORTDiscus, 422 from IEEE Xplore, and 13 from the ACM Digital Library, yielding 3,026 additional records. Within the supplemental set, 603 duplicates were removed; 142 further records matched the original library by DOI and/or standardised title, leaving 2,281 new records for screening. Reference lists of included reports and relevant reviews were also checked. No language restrictions were applied, although all included reports were published in English. Complete database-specific strategies are reported in **Supplementary Table S1**. The completed PRISMA 2020 checklist is provided as a separate supplementary file.

### 2.4 Selection process and report retrieval

Zhiyu Guan and Zicheng Jin independently screened titles and abstracts and then independently assessed potentially eligible full-text reports. Disagreements were resolved through discussion, with Chuangui Mao acting as the third author for adjudication when needed. Records were taken forward to full-text assessment whenever eligibility could not be determined from the title and abstract. One primary reason for exclusion was recorded for each excluded full-text report.

One report could not be retrieved despite searches of the available links and websites. It was recorded as not retrieved, and no eligibility or DPV judgement was made from its title or abstract alone.

### 2.5 Report, study, and analysis-unit structure

We used three related units of description. A report was an individual publication or full report. A study represented the underlying dataset, cohort, competition sample, or model-development investigation and could be described in more than one report. An analysis unit represented a distinct prediction task when the target, intended decision time, predictor cutoff, validation interpretation, or deployment boundary differed enough to require a separate assessment.

Multiple algorithms evaluated with the same target, predictors, decision time, and evaluation design were treated as one analysis unit. Separate analysis units were created only for substantively different tasks. Seven reports contributed two analysis units, and three study clusters contained more than one related report; these reports and clusters are identified in **Supplementary Table S3**. Related reports were consolidated at the study level for descriptive summaries and were not counted as independent replications.

The 80 included reports represented 77 underlying studies and contributed 87 eligible analysis units. Twenty-one analysis units came from the original search and 66 from the supplemental searches. All 87 units underwent DPV assessment, and the 78 Core and Comparator units also underwent formal PROBAST assessment. Reports that were not retrieved or were excluded after full-text assessment did not contribute analysis units. The manuscript specifies whether each result refers to reports, studies, or analysis units.

### 2.6 Data extraction and verification

Data were entered into a structured extraction form and independently checked against the full source reports by Zhiyu Guan (ZG) and Zicheng Jin (ZJ). The form covered study and sample characteristics, sport and competition level, prediction target and observation unit, intended user and decision, prediction time and predictor cutoff, modelling and tuning methods, validation design, performance measures, probability calibration or task-appropriate continuous-outcome reliability, missing-data handling, interpretability analyses, evidence layer, and reported deployment context or limitations. Study authors were not contacted for additional information. Items that were absent from the source report were recorded as Not reported.

ZG and ZJ independently verified the extracted data and DPV judgements against the source reports. Disagreements were adjudicated by CM, the third author, using the source report and both initial judgements. **Supplementary Table S7** records the verification and adjudication process; final DPV categories are reported in S3 and S4 and PROBAST assessments in S6.

Pre-adjudication agreement between ZG and ZJ was calculated for each of the six DPV domains and for the overall four-level classification using exact agreement and Cohen’s κ. A linearly weighted κ was also calculated for overall DPV because the categories were ordinal. Agreement methods followed established guidance for agreement studies and structured prediction-model appraisal (Kottner et al., 2011; Langenhuijsen et al., 2023). Paired rating distributions and agreement statistics are provided in **Supplementary Table S8B**.

Uncertainty was estimated using 10,000 non-parametric bootstrap resamples at the study level, with all analysis units from the same study retained within each resample. Percentile 95% confidence intervals were calculated for Cohen’s kappa and the weighted overall kappa, thereby accounting for dependence amongst analysis units originating from the same study (Vanbelle, 2019).

### 2.7 Task taxonomy and evidence layers

Analysis units were grouped as pre-game match prediction, in-game prediction or decision support, training-status or physiological monitoring, other eligible prediction tasks, post-event or high-leakage Comparators, or Boundary evidence. Core units addressed prediction or monitoring tasks central to the review question. Comparator units used information that became available after the relevant decision, complete-event data, future-derived information, or other inputs that prevented the intended pre-decision use; they were retained to show methodological contrasts. Boundary units represented related team-sport machine-learning applications outside the main prediction and monitoring tasks. Comparator and Boundary evidence were summarised separately from the Core findings.

### 2.8 PROBAST assessment

PROBAST was used alongside DPV to assess risk of bias and applicability. It was applied to all 78 Core and Comparator analysis units using its qualitative domain and overall judgements. The nine Boundary units were retained for contextual synthesis and were not formally assessed with PROBAST.

PROBAST was applied at the analysis-unit level because prediction tasks within the same report could differ in target, timing, predictors, and validation design. Risk of bias was judged against each study’s own prediction or retrospective target. The participants domain considered how athletes, teams, matches, or seasons were selected and whether the source data represented the eligible population. The predictors domain considered how predictors were defined and measured and whether their assessment was independent of the outcome. The outcome domain considered outcome definition and measurement, predictor independence, and the interval between prediction and outcome. The analysis domain covered effective sample size, missing data, repeated or clustered observations, feature selection, hyperparameter tuning, overfitting, validation, calibration or reliability assessment, and test-set independence. Random record splitting was a major concern when it failed to preserve the dependence or temporal structure required by the intended use. Preprocessing performed outside the training folds was treated as information leakage. Predictor availability at the decision time defined by this review was evaluated under applicability and DPV; this issue alone did not produce a High predictor-domain risk-of-bias rating.

Overall risk of bias was rated High when any domain was High, Low when all domains were Low, and Unclear otherwise. Applicability was assessed separately against the review question of deployable prediction in elite and high-level team sports. Comparator units therefore had High applicability concern when post-event or future-derived predictors were incompatible with the pre-decision use considered in this review, even if the original retrospective analysis had Low or Unclear internal risk of bias. ZG and ZJ independently assessed the PROBAST fields, and CM adjudicated disagreements. **Supplementary Table S6** provides the domain-specific rationales, whilst **Supplementary Table S7** documents the verification and adjudication record.

### 2.9 Deployable prediction validity

DPV described how well model development and evaluation supported a specified prediction or monitoring use. Its six dimensions were prediction timing, predictor availability, leakage control, validation strategy, calibration or task-appropriate reliability, and deployment boundary. The framework integrated established principles from prediction-model appraisal: specification of intended use and predictor applicability (Moons et al., 2014; Wolff et al., 2019; Moons et al., 2025), protection against information leakage and model-selection bias (Varma and Simon, 2006; Kaufman et al., 2012; Luo et al., 2016), validation aligned with the form of generalisation required in practice (la Roi-Teeuw et al., 2024), and assessment of calibration or task-appropriate predictive reliability (Steyerberg et al., 2010; Alba et al., 2017; Van Calster et al., 2019). These principles were organised around the decision time and deployment boundary of each task. DPV was applied as a structured qualitative judgement rather than a numerical score. **Figure 1** shows the framework and **Table 1** summarises the dimensions.

**Figure 1:**
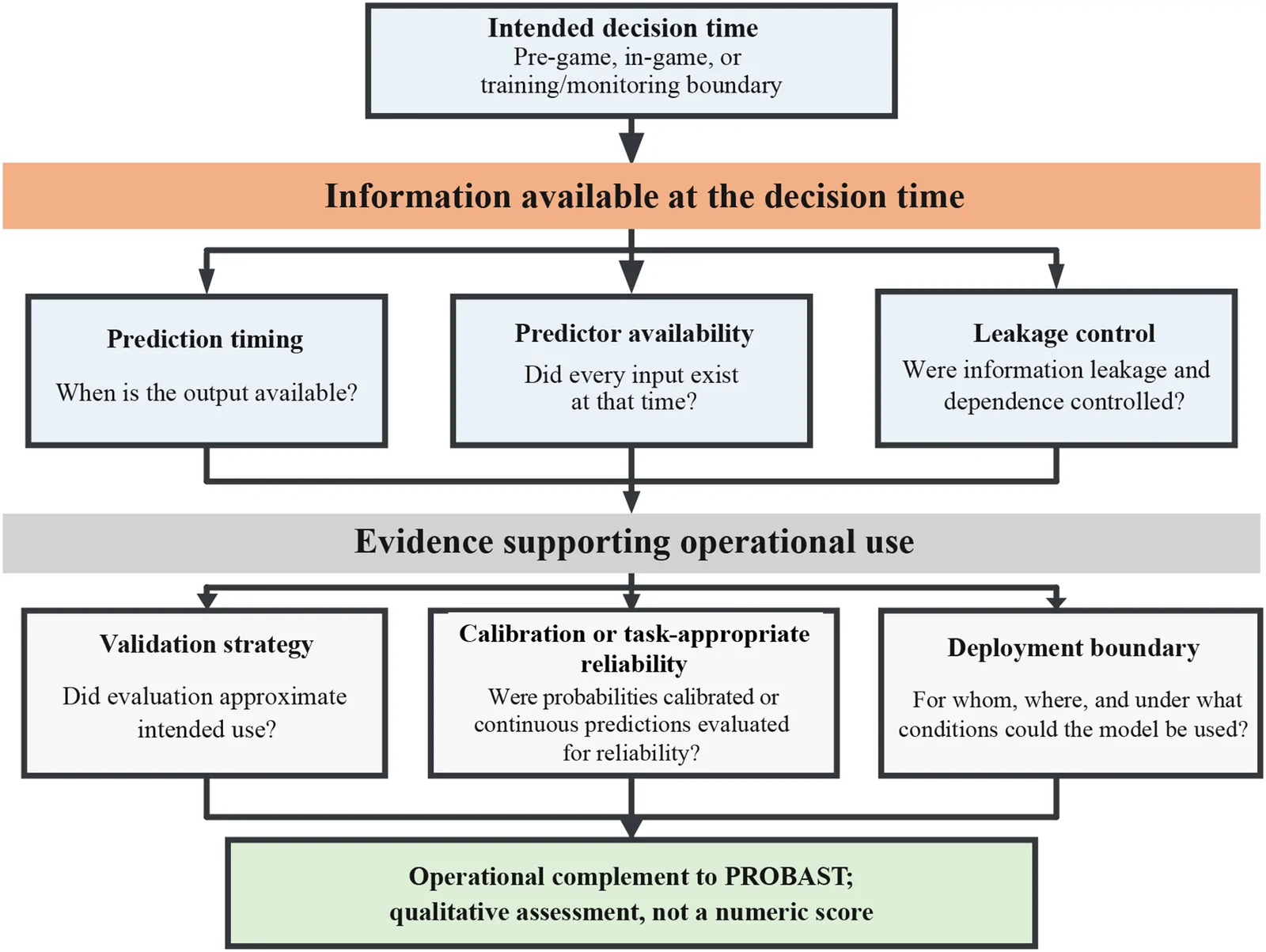
Conceptual framework for deployable prediction validity. The six dimensions assess prediction timing, predictor availability, leakage control, validation strategy, calibration or task-appropriate reliability, and deployment boundary in relation to the intended decision time. The framework complements PROBAST and uses qualitative, non-numerical ratings.

**Table 1:** Operational dimensions of deployable prediction validity.

Dimension	Operational question	Evidence supporting a favourable rating	Common concerns
Prediction timing	When is the output available?	Output available before or at the stated decision boundary.	Target already occurred; timing unclear; post-event estimation presented as forecasting.
Predictor availability	Did every input exist at that time?	Predictors frozen before the target decision or outcome horizon.	Complete-event or future-derived inputs; target-session measurements unavailable at the stated cutoff.
Leakage control	Were information leakage and dependence controlled?	Train-only preprocessing, feature selection, model selection, and tuning; linked observations protected from information sharing across evaluation boundaries.	Evaluation information influences preprocessing, feature or model selection, tuning, or threshold choice; linked observations create information sharing across a boundary intended to be independent.
Validation strategy	Did evaluation approximate intended use?	Temporal, grouped, or independently collected evaluation data that reflect the intended form of future use or generalisation.	Evaluation structure does not represent the intended future period, athlete, team, match, or other required generalisation; independence or grouping is inadequate for the stated use.
Calibration or task-appropriate reliability	Were probabilities calibrated or continuous predictions evaluated for reliability?	Sufficient: direct probability calibration or task-appropriate assessment of agreement, systematic error, residual patterns, or interval coverage.	Some concerns: a proper probability score without direct calibration diagnostics, or a limited agreement assessment. Not reported: discrimination or aggregate point-error metrics alone.
Deployment boundary	For whom, where, and under what conditions could the model be used?	Intended user, population, decision, data requirements, updating rule, and main limitations described.	User or action unclear; transportability assumed without evidence; retrospective analysis presented as operational decision support.

For probabilistic outcomes, calibration was assessed using reported comparisons between predicted and observed probabilities, including calibration plots, calibration intercepts or slopes, and reliability diagrams. Brier scores and ranked probability scores were treated as measures of overall probabilistic prediction quality (Gneiting and Raftery, 2007). When these scores were reported without direct calibration diagnostics, they provided partial evidence for this domain. For continuous outcomes, relevant evidence included agreement between predicted and observed values, systematic error, residual patterns across the prediction range, and prediction-interval coverage.

Ratings for the calibration or task-appropriate reliability domain reflected how completely each analysis unit assessed this aspect of performance. Sufficient indicated an adequate evaluation of probability calibration or continuous-outcome agreement for the prediction task. Some concerns indicated partial evidence, such as a proper probability score without direct calibration analysis or a limited assessment of agreement or residual error. Not reported was used when the analysis unit provided only discrimination, classification accuracy, or aggregate prediction error. Not applicable was reserved for prediction targets for which this type of reliability assessment was not relevant. The evidence supporting each rating is reported in **Supplementary Table S4**.

Overall DPV was assigned hierarchically according to the intended use, population, prediction horizon, predictor cutoff, and the methodological consequence of unresolved limitations. Non-deployable was used when information needed for prediction was unavailable at the intended decision time. Limited was used when timing was plausible but important problems in leakage control, dependence, or evaluation design could materially affect reported performance. Moderate indicated that decision-time inputs and evaluation were broadly appropriate, but uncertainty remained about model development, evaluation independence, or task-specific reliability. Moderate-to-strong was reserved for analysis units with clear evidence that predictors were available at the intended decision time, major sources of leakage were controlled, the final evaluation represented the intended use, and task-appropriate calibration, reliability, or probabilistic prediction-quality evidence was available. Overall classification reflected the materiality of remaining limitations for the specified use rather than the number of favourable or unfavourable domain labels. Accuracy, AUC, and R-squared were not used as thresholds for assigning DPV categories. Operational criteria were specified at both domain and overall-category levels to support consistent application across tasks (Kaiser et al., 2023).

Validation strategy assessed whether the evaluation structure represented the form of generalisation required in practice, including later sessions in the same athlete, new athletes, later matches, or another team (la Roi-Teeuw et al., 2024). Leakage control assessed whether evaluation information influenced preprocessing, feature selection, model selection, hyperparameter tuning, threshold choice, or other data-dependent development steps (Varma and Simon, 2006). Detailed criteria and examples are provided in **Supplementary Table S8A**.

### 2.10 Synthesis and reporting bias

Because the included studies varied widely in sport, prediction target, outcome scale, decision time, predictor structure, data source, validation design, and performance metric, we used a structured narrative and task-specific synthesis. Accuracy, area under the curve, F1 score, precision, recall, mean absolute error, root mean square error, R-squared, Brier score, ranked probability score, expected calibration error, and related measures were described within compatible tasks. We did not pool these metrics or use them to rank algorithms across different tasks.

The principal descriptive proportion was the proportion of 55 Core units rated at least Moderate; Comparator and Boundary evidence was summarised separately. To examine how this proportion changed when additional evidence was required, we conducted *post hoc* sensitivity analyses using three conditions without reassigning the four DPV categories: (1) Sufficient validation; (2) at least partial task-appropriate reliability or probability-quality evidence, defined as Sufficient or Some concerns; and (3) both conditions together. **Supplementary Table S8C** reports the resulting proportions.

Potential selective reporting was assessed qualitatively from descriptions of model selection, preprocessing, calibration, uncertainty, and independent evaluation. Funnel plots and small-study-effect tests were not appropriate because the included tasks did not share a common performance estimand. A separate GRADE certainty assessment was not undertaken because the review did not evaluate a common model, outcome, and use setting across studies (Foroutan et al., 2022). Figure source data are provided in **Supplementary Data S1**.

## 3 Results

### 3.1 Study selection and evidence structure

The combined searches identified 10,176 records. After 2,306 duplicates were removed, 7,870 records were screened and 7,699 were excluded at title and abstract stage. Full text was sought for 171 reports; one report could not be retrieved. Of 170 reports assessed for eligibility, 90 were excluded with reasons and 80 reports were included. The included reports represented 77 underlying studies and contributed 87 eligible analysis units. All 87 eligible units were assessed for deployable prediction validity. Reports not retrieved and reports excluded after full-text assessment did not constitute eligible analysis units. The selection process is shown in **Figure 2**, and report-level exclusion reasons are listed in **Supplementary Table S2**.

**Figure 2:**
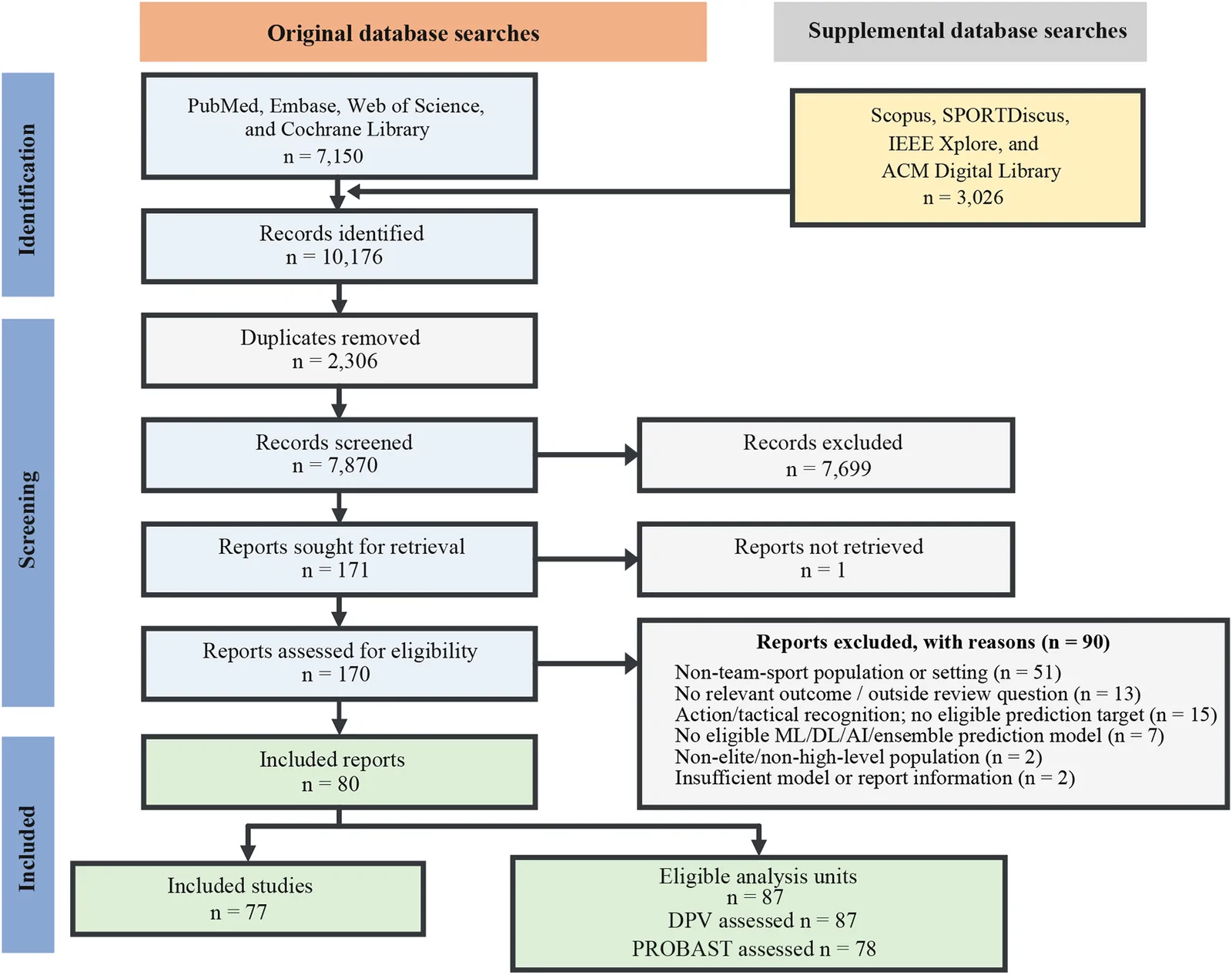
PRISMA 2020 flow diagram. The combined searches identified 10,176 records; 2,306 duplicates were removed, 7,870 records were screened, 171 reports were sought, one report was not retrieved, 170 reports were assessed, and 80 reports representing 77 studies were included. The evidence structure comprised 87 eligible analysis units; all 87 were assessed for deployable prediction validity and 78 were assessed with PROBAST.

### 3.2 Characteristics and evidence layers

The 80 included reports were published between 2016 and 2026. Twenty reports were identified through the original searches and 60 through the supplemental searches; these contributed 21 and 66 analysis units, respectively. Soccer accounted for 44 reports and basketball for 16. The remaining reports addressed Australian football (n=4), American football (n=3), cricket (n=3), volleyball (n=2), handball (n=2), baseball (n=2), Gaelic football (n=1), Canadian football (n=1), rugby sevens (n=1), and rugby union (n=1). Forty-nine reports contributed only Core units, 21 contributed only Comparator units, nine contributed Boundary units, and one report contributed both Core and Comparator units. At analysis-unit level, the corresponding counts were 55 Core, 23 Comparator, and nine Boundary units. Detailed report, study, analysis-unit, model, and performance characteristics are presented in **Supplementary Table S3**.

### 3.3 Verification and inter-rater agreement

In the full verification record, 69 of 87 analysis units had no field requiring adjudication and 18 had at least one disputed field (**Supplementary Table S7**). DPV-specific pre-adjudication agreement was assessed separately by domain and overall classification.

Pre-adjudication agreement was 87/87 (100%; Cohen’s κ = 1.000) for prediction timing, predictor availability, leakage control, validation strategy, and deployment boundary. Agreement for calibration or task-appropriate reliability was 75/87 (86.2%; κ = 0.697, 95% CI 0.578–0.818). Overall DPV agreement was 85/87 (97.7%; κ = 0.968, 95% CI 0.921–1.000), with a linearly weighted κ of 0.978 (95% CI 0.947–1.000). The two disagreements in the overall classification were between Moderate and Moderate-to-strong and occurred amongst units with disagreement in the reliability domain; neither crossed the ≥Moderate threshold. Full paired results are provided in **Supplementary Table S8B**.

### 3.4 PROBAST risk of bias and applicability

PROBAST was formally applied to 78 Core and Comparator analysis units. Overall risk of bias was High for 56 units (71.8%), Unclear for 21 (26.9%), and Low for one (1.3%). The analysis domain had the same distribution. Predictor risk of bias, judged against each study’s own prediction target, was High for three units, Unclear for 28, and Low for 47. The participants domain was Low risk in 65 units and Unclear in 13, whilst the outcome domain was Low risk in 72, Unclear in five, and High in one.

Overall applicability concern was Low for 49 units, Unclear for five, and High for 24. All 23 Comparator units had High applicability concern because their predictors were incompatible with the pre-decision use considered in this review. Their internal risk of bias was assessed separately: 21 were High, one Unclear, and one Low. Amongst pre-game units, 11 of 25 were High risk and 14 Unclear; amongst in-game units, nine of 11 were High and two Unclear. Training-status or physiological monitoring units included 12 High-risk and three Unclear-risk units. The four formally assessed other eligible tasks included three High-risk and one Unclear-risk unit. **Supplementary Table S6** provides the full domain-specific risk-of-bias and applicability judgements. **Figure 3** and **Table 2** summarise the task-specific distributions.

**Figure 3:**
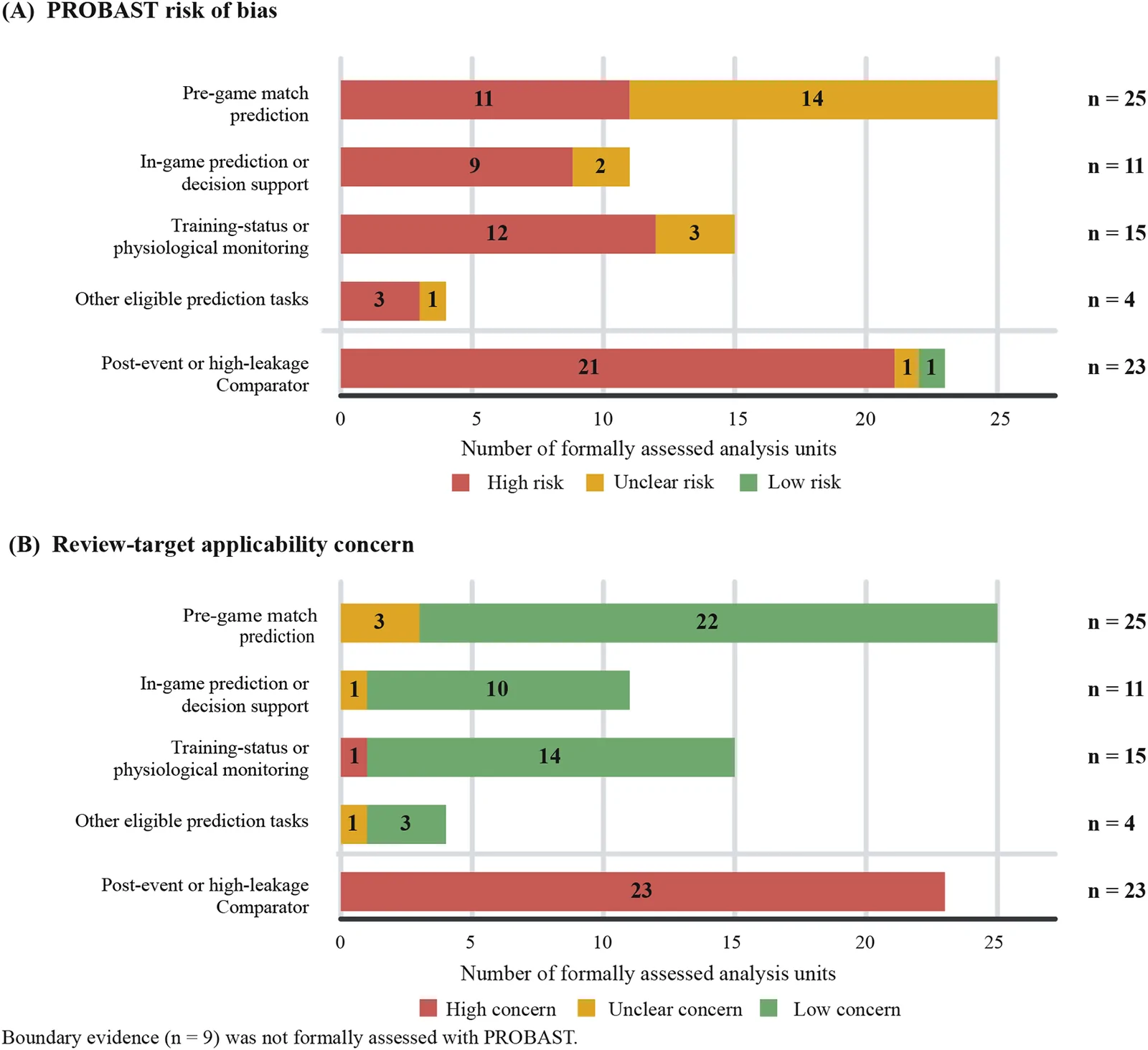
PROBAST risk of bias and applicability to the review question by task across 78 formally assessed Core and Comparator analysis units. **(A)** shows overall risk of bias: 56 High, 21 Unclear, and one Low. **(B)** shows overall applicability concern: 24 High, five Unclear, and 49 Low; all 23 Comparator units had High applicability concern. Boundary evidence was not formally assessed.

**Table 2:** Task- and evidence-layer-specific distribution of DPV classifications, PROBAST risk of bias, and high applicability concern.

Task/evidence group	Units	Moderate-to-strong	Moderate	Limited	Non-deployable	PROBAST ROB H/U/L	High applicability concern
Pre-game match prediction	25	4	10	11	0	11/14/0	0
In-game prediction or decision support	11	1	3	7	0	9/2/0	0
Training-status or physiological monitoring	15	0	6	9	0	12/3/0	1
Other eligible prediction tasks	4	0	2	2	0	3/1/0	0
Core total (primary denominator)	55	5	21	29	0	35/20/0	1
Post-event or high-leakage Comparator	23	0	0	0	23	21/1/1	23
Boundary evidence	9	0	1	2	6	Not assessed	Not assessed
All layers (descriptive)	87	5	22	31	29	56/21/1 (n=78)	24

Boundary evidence was not formally assessed with PROBAST.

### 3.5 Deployable prediction validity across tasks

Of the 55 Core units, five were Moderate-to-strong, 21 Moderate, and 29 Limited. The principal descriptive proportion was 26/55 (47.3%) rated at least Moderate. All 23 Comparator units were Non-deployable for the pre-decision use defined in this review. The nine Boundary units included one Moderate, two Limited, and six Non-deployable units. Across all 87 units, five were Moderate-to-strong (5.7%), 22 Moderate (25.3%), 31 Limited (35.6%), and 29 Non-deployable (33.3%). **Table 2** and **Figure 4** show the task- and layer-specific distributions, and **Supplementary Table S4** provides unit-level evidence.

**Figure 4:**
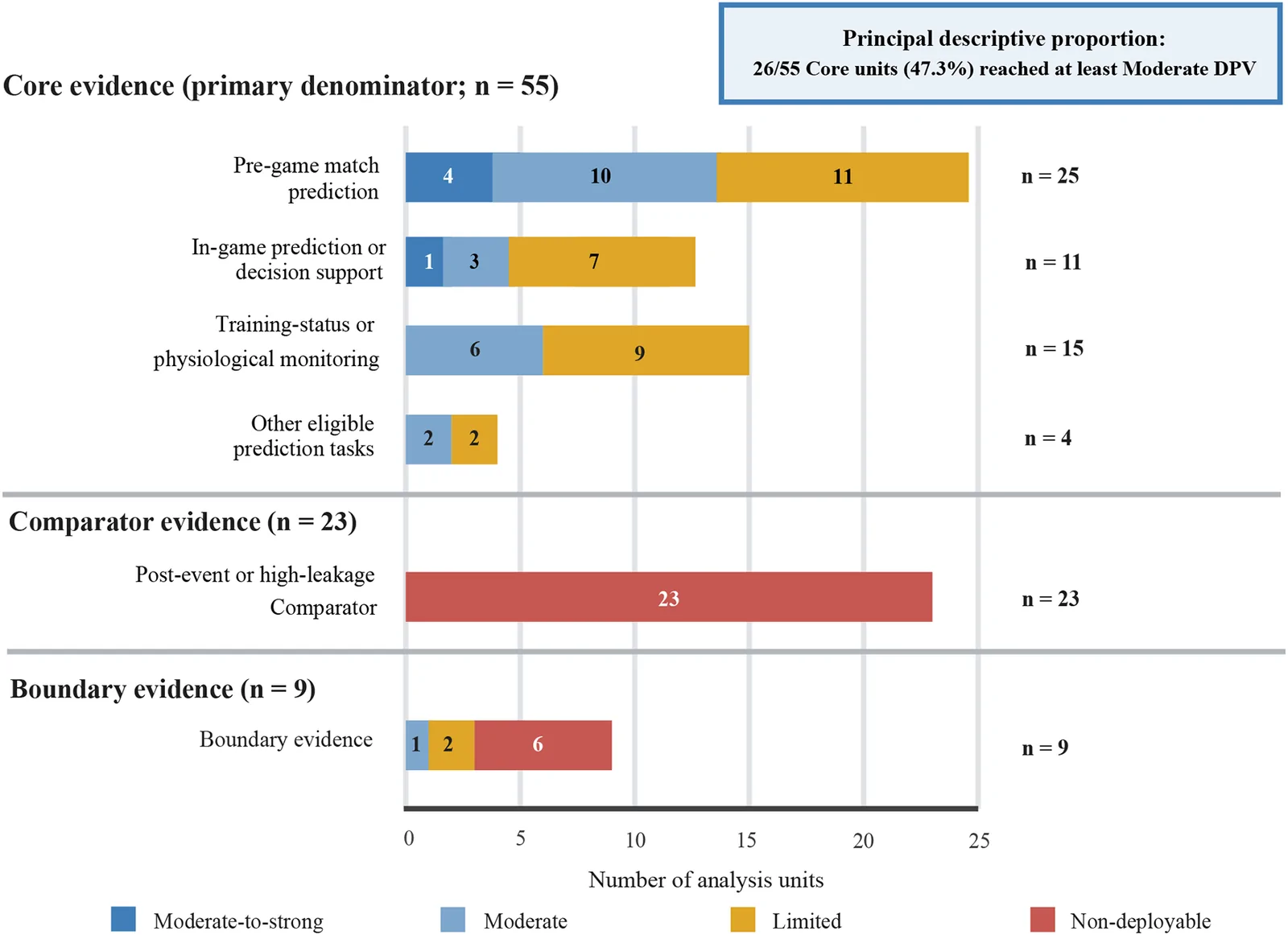
Task- and evidence-layer-specific distribution of deployable prediction validity across 87 analysis units. The 55 Core units comprised five Moderate-to-strong, 21 Moderate, and 29 Limited units; 26/55 (47.3%) reached at least Moderate DPV, the principal descriptive proportion. All 23 Comparator units were Non-deployable for the pre-decision use defined in this review. The nine Boundary units comprised one Moderate, two Limited, and six Non-deployable units.

In the *post hoc* sensitivity analyses, 19/55 (34.5%) Core units met the primary DPV threshold together with Sufficient validation, 14/55 (25.5%) met the threshold and had at least partial task-appropriate reliability or probability-quality evidence, and 9/55 (16.4%) met both additional conditions. These values were 12.7, 21.8, and 30.9 percentage points below the principal descriptive proportion, respectively. Of the 26 Core units rated at least Moderate, seven did not have Sufficient validation, 12 had no reported task-appropriate reliability or probability-quality evidence, and two had both limitations. **Supplementary Table S8C** provides the unit-level sensitivity analysis.

Prediction timing was Sufficient in 57 units and predictor availability in 55. Leakage control was Sufficient in seven units, Some concerns in 31, and High concerns in 49. Validation strategy was Sufficient in 21 units, Some concerns in 41, and High concerns in 25. Calibration or task-appropriate reliability was Sufficient in six units, Some concerns in 16, Not reported in 64, and Not applicable in one. Deployment boundary was Clear in 23 units, Conditional in 35, Retrospective only in 17, and Not deployable in 12. **Figure 5** and **Table 3** show the six domain-level distributions.

**Figure 5:**
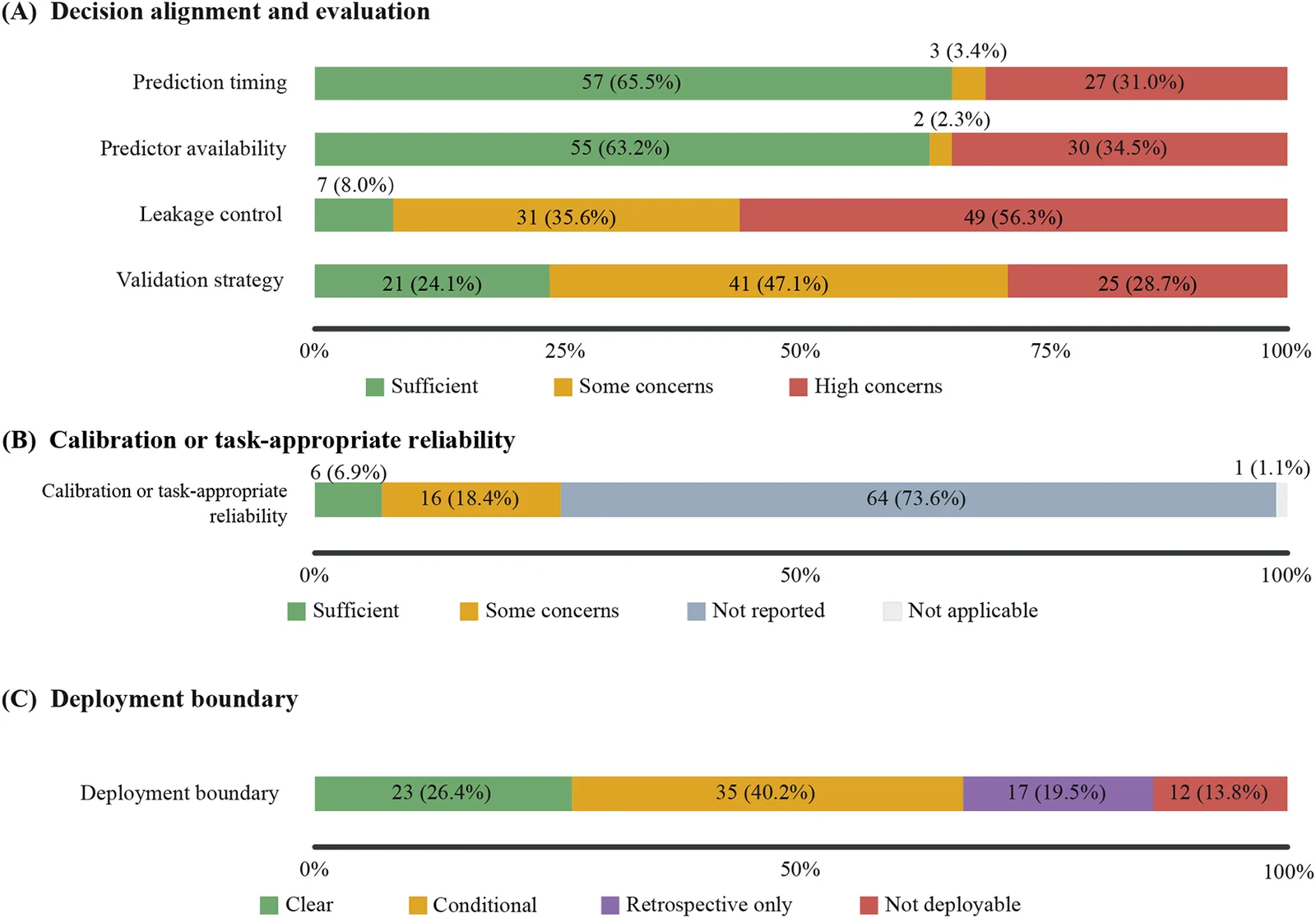
Distribution of ratings across the six deployable prediction validity dimensions for 87 analysis units. **(A)** Decision alignment and evaluation, including prediction timing, predictor availability, leakage control, and validation strategy. **(B)** Calibration or task-appropriate reliability. **(C)** Deployment boundary. Leakage control was rated Sufficient in seven units, Some concerns in 31, and High concerns in 49. Validation strategy was rated Sufficient in 21 units, Some concerns in 41, and High concerns in 25. Calibration or task-appropriate reliability was rated Sufficient in six units and Some concerns in 16; it was Not reported in 64 and Not applicable in one. Deployment boundary was Clear in 23 units, Conditional in 35, Retrospective only in 17, and Not deployable in 12.

**Table 3:** Distribution of the six deployable prediction validity dimensions across 87 analysis units.

Dimension	Favourable/sufficient	Intermediate/partial	High concerns/Not reported/Not applicable/Not deployable
Prediction timing	57 Sufficient	3 Some concerns	27 High concerns
Predictor availability	55 Sufficient	2 Some concerns	30 High concerns
Leakage control	7 Sufficient	31 Some concerns	49 High concerns
Validation strategy	21 Sufficient	41 Some concerns	25 High concerns
Calibration or task-appropriate reliability	6 Sufficient	16 Some concerns	64 Not reported; 1 Not applicable
Deployment boundary	23 Clear	35 Conditional	17 Retrospective only; 12 Not deployable

### 3.6 Pre-game match prediction

Fourteen of the 25 pre-game units reached at least Moderate DPV. Evaluation designs included later-fixture testing, held-out seasons, tournament holdouts, and internal resampling. Other reports in the pre-game task group included Kapadia et al. (2019), Beal et al. (2020), Horvat et al. (2020), Stübinger et al. (2020), Chen et al. (2021), Hsu (2021), Osken and Onay (2022), Choi et al. (2023), Głowania et al. (2023), Yeung et al. (2023), Alves and Barbosa (2025), Macrì Demartino et al. (2025), Ren et al. (2025), Wong et al. (2025), Zhong (2025), and Salaj (2026). Four units were Moderate-to-strong: Nashwan et al. (2026), the result-probability unit of Berrar et al. (2024); Fahey-Gilmour et al. (2019), and Constantinou (2019).

Berrar et al.’s exact-score unit was Moderate; it reported RMSE but no task-appropriate agreement analysis. The ordinal-foul unit of Pérez-Blanco and Salmerón (2026) was also Moderate. It used sequential evaluation and ranked probability scoring, but the report did not clearly separate discretisation selection from testing. The Esme and Kiran (2018) unit was rated Limited because the same 153 test matches informed the choice of k and the subsequent risk-filtering analysis.

### 3.7 In-game prediction or decision support

Four of the 11 in-game units reached at least Moderate DPV. Other reports in the in-game task group included Mohandas et al. (2023), Ouyang et al. (2024), Tyagi et al. (2024), Baadi et al. (2025), and Olaizola et al. (2025). Kayhan and Watkins (2018) provided Moderate-to-strong evidence for the principal score-and-clock snapshot model. Yao et al. (2022); Aarons et al. (2023), and the cumulative in-game unit of AlMulla et al. (2023) were Moderate. Common inputs included score, time remaining, possession, and accumulated match events.

Hill (2022) reported calibration plots and leave-one-season-out evaluation; the same procedure was used for grid-search tuning, with no separate outer or post-selection test, and the unit was Limited. AlMulla et al. (2023) used nested cross-validation, but match-level grouping of paired team records, temporal testing, and calibration assessment were not reported; the cumulative in-game unit was Moderate (**Supplementary Table S4**).

### 3.8 Training-status or physiological monitoring

The 15 training-status and physiological monitoring units covered perceived exertion, heart-rate response, wellness, fatigue, recovery, readiness, workload, and cardiovascular adaptation. Other reports in this task group included Jaspers et al. (2018), Perri et al. (2021), Kim et al. (2022), Marynowicz et al. (2022), Mandorino et al. (2023), Sheridan et al. (2024), Sharma et al. (2025), Simonelli et al. (2026a), and Simonelli et al. (2026b). Six reached at least Moderate DPV, and one had Sufficient leakage control. Examples included future wellness prediction (Op de Beéck et al., 2019; Rossi et al., 2022), modelling of heart-rate response and related monitoring indices (Mandorino et al., 2024; Diouron et al., 2025), and current recovery estimation (Simonelli et al., 2025).

Repeated measurements from the same athletes were common in this group. Evaluation designs included random record splits, temporal testing, and player-held-out testing. Several reports did not clearly describe separation of sessions or future periods or the timing of preprocessing. None of the 15 units was rated Moderate-to-strong.

### 3.9 Other eligible prediction tasks

Four units addressed other eligible tasks, including future workload forecasting and post-session perceived-exertion estimation (Vallance et al., 2023; Leppich et al., 2024; Carey et al., 2016; Felice, 2026). Two were Moderate and two Limited. None had Sufficient validation. One reported partial task-appropriate reliability, whereas three did not report this type of evidence. All four had Conditional deployment boundaries.

### 3.10 Post-event or high-leakage Comparators

All 23 Comparator units were classified as Non-deployable for the pre-decision use defined in this review (Martins et al., 2017; Thabtah et al., 2019; Young et al., 2019; Ballı and Özdemir, 2021; Dijkhuis et al., 2021; Filiz, 2023; Zhao et al., 2023; Song et al., 2024; Teixeira et al., 2024; Lima et al., 2025; Luo et al., 2025; Ming and Jin, 2025; Ou-Yang et al., 2025; Shah and Zaman, 2025; Ulloa-Díaz et al., 2025; Weirich et al., 2025; Bae and Chiu, 2026; Castellano et al., 2026; Dong et al., 2026; Karakoç et al., 2026; Kato et al., 2026). These models commonly used complete-match technical statistics, final season aggregates, same-session post-target variables, future rankings, target-derived baselines, or graph links containing future information. Because these predictors would not be available at the relevant decision time, every Comparator unit had High applicability concern. Internal risk of bias was assessed separately and was High in 21 units, Unclear in one, and Low in one. Comparator units were retained to show methodological contrasts and did not contribute evidence for prospective deployment. Unit-level rationales are provided in **Supplementary Table S5**.

### 3.11 Boundary evidence

Nine Boundary units were retained to represent related applications outside the main prediction and monitoring tasks. One was rated Moderate, two Limited, and six Non-deployable (Ali and Yilmaz, 2023; Raabe et al., 2023; Epp-Stobbe et al., 2025; Teixeira et al., 2025; Zhang et al., 2025; Górecki et al., 2026; Kim et al., 2026; Liu and Hong, 2026; Mohandas et al., 2026). These applications included tactical representation and possession-regain tasks, player-partnership assessment, long-term talent or ranking models, missing-value imputation, causal-effect exploration, and retrospective team-strength estimation. They were relevant to team-sport analytics, although their targets or use settings fell outside the main questions of this review. **Supplementary Table S5** provides the unit-level rationales.

## 4 Discussion

### 4.1 Principal findings

Evidence for deployable machine-learning prediction in team sports was uneven. Of the 55 Core units, 26 reached at least Moderate DPV and 29 were Limited. High or Unclear PROBAST ratings were also common.

The sensitivity analyses showed that the proportion meeting additional evidential conditions was lower than the principal descriptive proportion. Many Core units rated at least Moderate lacked Sufficient validation, reported task-appropriate reliability or probability-quality evidence, or both. Validation gaps mainly involved evaluation structures that did not clearly represent future periods or independent units, whereas reliability gaps mainly reflected limited direct calibration, probability-quality, or individual-agreement evidence.

Reliability evidence also varied in strength. Three of the five Moderate-to-strong units reported a proper probability score without direct calibration diagnostics. These scores assess overall probabilistic prediction quality but do not directly characterise calibration across the relevant probability range.

The main methodological limitations differed across tasks. Pre-game models often had a clear information cutoff because predictors could be fixed before competition. In-game models sometimes reported probability-quality measures, but repeated predictions within the same match and specialised data feeds created additional evaluation challenges. Training-status or physiological monitoring studies included both future forecasting and current-state estimation, so similar performance metrics could describe very different practical uses. Considering decision time and evaluation design alongside model performance therefore adds information that application-focused reviews may miss.

### 4.2 Interpreting reported performance in relation to intended use

Reported performance has meaning only in relation to the prediction target, the information available at prediction time, the data-generating process, and the evaluation design. Complete-match statistics may support retrospective classification, yet they are unavailable for a pre-game decision. Post-session perceived-exertion estimates can help complete monitoring records or describe response to a finished session, whereas forecasting a future session requires a different information cutoff and evaluation design. Stating the intended use before evaluating a model makes these distinctions easier to interpret.

Several design choices can make performance look better than it is likely to be in future use. Whole-dataset normalisation, imputation, feature selection, class balancing, or outcome-derived thresholding can allow evaluation data to influence model development. Choosing hyperparameters from evaluation folds creates a similar risk. Random record splitting is also problematic when related observations cross a boundary that should remain independent, such as the two team records from one match or future sessions contributing information to earlier predictions. Repeated measurements from the same athlete can still be appropriate for personalised monitoring when chronological ordering is preserved and all data-dependent processing is confined to information available before the relevant prediction.

Interpretability provides complementary information about a fitted model. SHAP values, feature importance, and partial-dependence analyses can show how predictions depend on model inputs. Their practical meaning is easier to judge once the prediction target, information cutoff, validation design, and deployment boundary are clear. Explainability on its own provides no evidence that predictors are available at the required time, probabilities are calibrated, associations are causal, or performance will transfer to a new setting.

### 4.3 Task-specific interpretation

#### 4.3.1 Pre-game match prediction

Pre-game forecasting offers a relatively clear information cutoff because predictors can be fixed before competition and later fixtures can be reserved for evaluation. In the challenge designs used by Berrar et al. (2024) and Constantinou (2019), predictions were fixed before outcomes in the test set were known. Fahey-Gilmour et al. (2019) kept the two team records from each match together during development and evaluated trained pipelines on a later season. These choices protect against different sources of optimism. If several candidate pipelines are repeatedly compared on the same test season, however, the reported performance of the selected winner may still be optimistic. For probability forecasts, direct calibration assessment complements proper scoring rules by showing how predicted probabilities correspond to observed outcomes.

#### 4.3.2 In-game prediction or decision support

In-game evaluation should represent new matches whilst preserving the information available at each point in a match. Later-season testing provided evidence of temporal transfer in some studies, whereas others relied on internal resampling or did not report match-level grouping of paired team records. Calibration or probability-quality assessment, data latency, and model-update timing remain important for live use.

#### 4.3.3 Training-status or physiological monitoring

Before training, a forecast can inform planning only if its inputs are available before the session decision. Op de Beéck et al. (2019) used preceding load and wellness to predict future perceived wellness, and Rossi et al. (2022) forecast next-day wellness from workload histories. These applications need a clearly defined prediction horizon and evaluation on later observations. Personalised models can use information from the same athlete when that information was collected before the predicted session. Simonelli et al.’s (2025) TQR model addressed a different task by estimating the athlete’s current morning recovery state. In that setting, co-measured fatigue and soreness are available inputs, whilst the added value beyond the questionnaire and the agreement of individual predictions remain relevant to practical use.

Within-session monitoring uses information generated during exercise to interpret the response observed up to that point. Mandorino et al. (2024) modelled heart-rate response to training drills and derived a fitness index from differences between measured and predicted heart rate; Diouron et al. (2025) tracked a related difference signal across a season. These studies illustrate physiological monitoring based on the same collection window as the external-load and heart-rate data. Real-time use would additionally require the relevant data window to be complete and processed quickly enough to inform the next decision. The meaning of the derived signal also depends on drill context, measurement consistency, and evidence that the index reflects the intended physiological construct.

After training, completed-session data can be used to estimate or impute perceived exertion, as in Vallance et al. (2023) and Leppich et al. (2024). Such estimates may help complete a monitoring record or describe an athlete’s response to the finished load. Their usefulness depends on prediction error across low and high exertion levels and on the consequences of replacing a reported value with an estimate. Fatigue, wellness, recovery, exertion, and cardiovascular response are distinct constructs measured over different time windows. Model outputs should therefore be interpreted alongside the training objective and the broader workload-response context (Drew and Finch, 2016; Gabbett, 2016; Windt and Gabbett, 2017; Vanrenterghem et al., 2017). Whether acting on these outputs improves training or athlete outcomes requires prospective evaluation.

#### 4.3.4 Other eligible tasks, comparators, and boundary evidence

The other eligible tasks concerned future-session workload forecasting and session-level perceived-exertion estimation. Their targets were generally coherent, but evidence of performance beyond the source sample was limited. Comparator units served a different role. By using complete-event statistics, future-derived features, or variables measured close to the target, they showed that strong retrospective performance can still have little relevance to an earlier decision. Reporting these units separately preserves that methodological lesson without treating them as evidence for prospective deployment.

Boundary evidence covered related applications such as tactical representation, partnership assessment, causal-effect exploration, long-term ranking, and imputation. These studies contribute to team-sport analytics, but their targets fall outside the main prediction and monitoring questions of this review. They were therefore summarised as contextual evidence rather than combined with the Core results.

### 4.4 Complementary roles of PROBAST and deployable prediction validity

PROBAST and DPV address related questions from different angles. PROBAST evaluates risk of bias and applicability across participants, predictors, outcomes, and analysis. In this review, its analysis domain captured concerns such as small effective samples, inadequate handling of repeated observations, non-nested model development, overfitting, incomplete validation, and absent or inadequate calibration. High and Unclear ratings were common, with only one Low-risk unit, so even the strongest deployment-oriented evidence requires cautious interpretation.

DPV adds an explicit decision-time perspective by asking when the output becomes available, what information each input requires, which future observations were used for evaluation, and what evidence supports prediction reliability. An analysis unit can therefore have acceptable internal risk of bias for a retrospective target whilst offering little evidence for an earlier prospective use. Using PROBAST and DPV together allows internal methodological quality and suitability for the intended use to be interpreted separately.

### 4.5 Implications for research and practice

Future studies should describe the intended user, decision, prediction time, eligible population, predictor cutoff, outcome horizon, updating schedule, and proposed action. The evaluation can then be designed around the relevant form of generalisation, such as later matches, later sessions in the same athlete, new athletes, or another team. The sampling unit and temporal order should match that intended use (la Roi-Teeuw et al., 2024). When both new individuals and future periods matter, grouped and chronological holdouts provide complementary evidence.

All data-dependent steps should be fitted within the development data, including imputation, scaling, threshold construction, feature selection, dimensionality reduction, class balancing, and tuning. Final performance should then be evaluated on an untouched test set or through an appropriately nested outer resampling design. Reports of temporal or new-population testing should describe the actual sampling and information boundaries. If a model is selected after repeated comparison on a test set, a separate final assessment is needed to estimate the performance of that selected model.

Probability models should report discrimination, a proper scoring rule, and direct calibration diagnostics. Continuous-outcome models should examine systematic error across the prediction range and, where relevant, prediction-interval coverage. Comparisons with simple historical averages, parsimonious statistical models, and current practice can help establish added value. The practical importance of an error depends on the decision and outcome scale, so a single accuracy threshold is unlikely to be meaningful across these tasks.

The evidence base also needs to move beyond internal model performance. Useful next steps include prospective silent evaluation, testing within real workflows, planned model updating, monitoring for performance drift, and evaluation of fairness, usability, cost, and decision impact. Operational testing should begin only after data requirements, likely failure modes, uncertainty, and limits of transportability have been described clearly.

### 4.6 Strengths and limitations

This review combined searches across biomedical, sport-science, engineering, and computing databases. Distinguishing reports, underlying studies, and analysis units allowed related publications and substantively different prediction tasks to be handled separately. Full-text assessment established predictor cutoffs and the timing of each intended decision, whilst the Core, Comparator, and Boundary layers kept different types of evidence separate in the synthesis. Two authors independently verified all 87 units against the source reports, with adjudication by a third author when needed.

Several limitations should be considered. The reported agreement reflects concordance between the two independent assessors before adjudication; independent application by other review teams would provide additional evidence on reproducibility across assessors. DPV ratings also depended on the detail reported in the source articles. When a method was not described, we could identify the resulting uncertainty but could not determine whether the method had been omitted in practice. Reuse of public datasets across otherwise separate studies may also create dependence beyond the study clusters considered in this analysis.

Publication and reporting bias could not be assessed statistically because the tasks and performance measures were too heterogeneous for a common analysis. Studies with positive or technically novel findings may be more likely to appear in the published literature, and incomplete reporting can obscure unsuccessful validation, poor calibration, or negative external tests. Consequently, the published evidence may present a more favourable picture of model performance than the complete set of conducted analyses.

The scope of this review was limited to methodological evidence supporting each intended use. It did not assess whether model-guided decisions improve athlete outcomes in practice. Prospective studies that embed prediction models in real workflows are therefore needed to establish decision impact and practical validity.

## 5 Conclusion

Evidence for deployable machine-learning prediction and monitoring in elite and high-level team sports remains uneven. Of 55 Core units, 26 (47.3%) reached at least Moderate DPV. In the *post hoc* sensitivity analyses, 9/55 (16.4%) met the primary DPV threshold together with both Sufficient validation and at least partial task-appropriate reliability or probability-quality evidence. This difference highlights the importance of validation and reliability evidence when interpreting reported performance for practice.

Decision-time predictor availability, leakage control, evaluation aligned with intended use, and task-appropriate reliability assessment are practical features to check when interpreting prediction performance. Even the strongest evidence identified in this review applies to the task and data setting in which it was tested. Comparator and Boundary evidence remain informative when interpreted within the decision times and use settings they actually address.

Future progress depends on clearer links between the sport decision, the prediction protocol, and the population in which performance is evaluated. DPV offers a structured way to examine these links alongside PROBAST. The effect of using a model to guide training, health, or performance decisions will ultimately need to be established in prospective impact studies.

## Data Availability

The original contributions presented in the study are included in the article/**Supplementary Material**. Further inquiries can be directed to the corresponding authors.
