# Supplementary material for: Deployability of machine-learning prediction models in elite and high-level team sports: a task-specific systematic review
Source: Front Physiol. 2026 Sep 30;17:1920214. doi: 10.3389/fphys.2026.1920214 (PMC13661197; doi:10.3389/fphys.2026.1920214)
Supplement: Supplementary file 1 [file Table2.docx]

**Supplementary Table S6. PROBAST risk-of-bias and applicability assessment**

Risk-of-bias and applicability judgements were independently verified by Zhiyu Guan and Zicheng Jin against the complete source reports. Disagreements were adjudicated by Chuangui Mao, the third author. Supplementary Table S7 documents the verification and adjudication record, including source locations and adjudication decisions. Core and Comparator units were formally assessed (n=78); Boundary units (n=9) were retained for contextual synthesis but were not formally assessed.

Internal risk of bias and review-target applicability are reported separately. Predictor unavailability at the review decision point is an applicability concern and does not, by itself, establish high internal risk of bias for the study’s original target. Overall ROB among assessed units: 56 high, 21 unclear and 1 low. All 23 Comparator units retained high overall applicability concern.

**Part A. Risk-of-bias judgements and principal rationale**

| **Study / analysis unit** | **Layer** | **Participants ROB** | **Predictors ROB** | **Outcome ROB** | **Analysis ROB** | **Overall ROB** | **Principal ROB rationale** | **Source page / section** |
| --- | --- | --- | --- | --- | --- | --- | --- | --- |
| Zhong (2025) | Core | Unclear | Unclear | Low | Unclear | Unclear | No domain was high, but Participants, Predictors, Analysis remained unclear because reporting or safeguards were insufficient. Principal basis: Repeated random, sequential and cross-season evaluations were reported; cross-season testing is temporal internal validation rather than external validation. Target-game information was excluded, but complete nesting of preprocessing and model selection was not fully documented. | Source report, pp. 6 and 12–16; dataset, pre-game feature construction, train-test evaluation and model-comparison results |
| Ouyang et al. (2024) — first two quarters / first half-related in-game model | Core | Unclear | Unclear | Low | High | High | High overall ROB because the Analysis domain was high. Principal basis: Feature selection, optimization and comparison of multiple algorithms were not shown to be fully nested within resampling. Ten-fold cross-validation was reported, but no independent temporal holdout was used. | Source report, pp. 4 and 7–14; algorithm, data processing, second-quarter predictor set, cross-validation and in-game results |
| Ouyang et al. (2024) — first three quarters-related in-game model | Core | Unclear | Unclear | Low | High | High | High overall ROB because the Analysis domain was high. Principal basis: Feature selection, optimization and comparison of multiple algorithms were not shown to be fully nested within resampling. Ten-fold cross-validation was reported, but no independent temporal holdout was used. | Source report, pp. 4 and 7–15; algorithm, data processing, third-quarter predictor set, cross-validation and in-game results |
| Ou-Yang et al. (2025) | Comparator | Unclear | Low | Low | High | High | Complete match statistics were measured objectively for the study’s retrospective explanatory target. High overall ROB is retained because random/repeated splits and model selection do not address dependence and overfitting; temporal inapplicability is recorded only under applicability. | Source report, pp. 4 and 7–9; dataset, model development, cross-validation and performance results |
| Yao et al. (2022) | Core | Unclear | Low | Low | Unclear | Unclear | No domain was high, but Participants, Analysis remained unclear because reporting or safeguards were insufficient. Principal basis: Internal evaluation was reported, while strict forward temporal independence was not fully demonstrated. No direct future-outcome predictor was used, but preprocessing and model-selection boundaries were incompletely reported. | Source report, pp. 7 and 9–11; Chinese Super League data, evaluation, Ranked Probability Score and calibration analyses |
| Kayhan and Watkins (2018) | Core | Unclear | Unclear | Low | Unclear | Unclear | No domain was high, but Participants, Predictors, Analysis remained unclear because reporting or safeguards were insufficient. Principal basis: Earlier seasons were used for development and the subsequent 2016-2017 season was used for temporal testing. The main snapshot model is temporally valid, while some expanded covariates have training-deployment availability concerns. | Source report, pp. 4–5 and 10–16; dataset, snapshot construction, independent validation season, Brier scores and reliability analyses |
| Song et al. (2024) | Comparator | Unclear | Low | Low | High | High | Predictors are ordinary completed-match variables for the reported retrospective target. High overall ROB is retained because of the small data set and non-grouped random validation; post-event timing remains a high applicability concern. | Source report, pp. 3 and 5–7; machine-learning methods, training-validation split, cross-validation and model-performance results |
| Osken and Onay (2022) | Core | Unclear | Unclear | Low | High | High | High overall ROB because the Analysis domain was high. Principal basis: The main feature definitions incorporate future or target-game information; only the robustness analysis substitutes historically available values. The main evaluation used a random 75/25 split, while a stricter robustness analysis held out the 2017-2018 season. | Source report, pp. 5 and 7–10; player/game data, prediction inputs, random split, held-out-season robustness check and results |
| Zhao et al. (2023) | Comparator | Unclear | Unclear | Low | High | High | Predictor construction through the graph representation is insufficiently separated from the full data structure. High analysis ROB is retained because global representation/feature construction may transmit information across partitions. | Source report, pp. 4–5 and 8–11; graph construction, dataset, feature engineering and 70/10/20 evaluation |
| Weirich et al. (2025) | Comparator | Unclear | Low | Low | High | High | Predictor measurement itself is not high risk for the study’s retrospective target. High analysis ROB remains because preprocessing/tuning and model comparison were not demonstrably isolated from evaluation, including reuse across random and chronological analyses. | Source report, pp. 2–7; dataset, preprocessing, model architecture, random evaluation and chronological forecasting evaluation |
| Ming and Jin (2025) | Comparator | Unclear | Low | Low | High | High | Completed-match predictors are acceptable for the original target. Feature analysis and preprocessing preceded repeated split comparisons, and test proportions were used in model comparison; therefore high analysis and overall ROB remain. | Source report, pp. 3, 6 and 8–12; sample, standardisation, data splitting, cross-validation, test results and external-validation description |
| Macrì Demartino et al. (2025) | Core | Low | Low | Low | Unclear | Unclear | No domain was high, but Analysis remained unclear because reporting or safeguards were insufficient. Principal basis: Models trained on historical matches were evaluated on later tournament matches outside the estimation data. The tournament separation is credible, but the report did not describe every preprocessing and updating step in enough detail to determine whether all such steps were confined to the development data. | Source report, pp. 10-17; Sections 4-5 |
| Zhang et al. (2025) | Boundary | Not assessed | Not assessed | Not assessed | Not assessed | Not assessed | Not formally assessed because the unit was retained as boundary evidence and was excluded from the formal PROBAST synthesis. | Source report, pp. 3-11; Methods and Results |
| Mohandas et al. (2026) | Boundary | Not assessed | Not assessed | Not assessed | Not assessed | Not assessed | Not formally assessed because the unit was retained as boundary evidence and was excluded from the formal PROBAST synthesis. | Source report, pp. 7-12 and 27-31; Sections 3.1-3.6, 4.8, and 5 |
| Raabe et al. (2023) | Boundary | Not assessed | Not assessed | Not assessed | Not assessed | Not assessed | Not formally assessed because the unit was retained as boundary evidence and was excluded from the formal PROBAST synthesis. | Source report, pp. 11-15; Sections 4.1-4.6 |
| Mandorino et al. (2024) | Core | Low | Low | Low | High | High | High overall ROB because the Analysis domain was high. Principal basis: Feature selection and tuning used a separate 20% subset, but repeated records from the same players were not separated by player or time. Five-fold internal cross-validation was used without player-grouped, squad-grouped, or chronological validation. | Source report, pp. 1-3; Methods: ML models, preprocessing, RFE, tuning, and cross-validation |
| Leppich et al. (2024) | Core | Low | Unclear | Low | High | High | High overall ROB because the Analysis domain was high. Principal basis: The dataset was shuffled and repeated observations from the same players could occur in both training and validation folds; fold-contained preprocessing was not fully demonstrated. Five-fold internal cross-validation was reported, but no player-level or temporal holdout assessed performance for new players or later periods. | Source report, pp. 1, 3, and 5-6; Training and experimental setup |
| Vallance et al. (2023) | Core | Low | Unclear | Low | High | High | High overall ROB because the Analysis domain was high. Principal basis: Whole-dataset normalization and player-mean imputation were performed before random cross-validation. Repeated ten-fold cross-validation was internal and random, without holding out players or future periods. | Source report, pp. 1-3; Methods |
| Diouron et al. (2025) | Core | Low | Low | Low | High | High | High overall ROB because the Analysis domain was high. Principal basis: Initial model comparison used repeated random splits, while the final monitoring stage used a more appropriate expanding weekly structure. The expanding weekly procedure is temporally ordered, but it remained an internal single-club evaluation and model selection was not independently nested. | Source report, pp. 1 and 4-8; machine-learning methods and delta-HR construction |
| Ulloa-Díaz et al. (2025) | Comparator | Low | Low | Low | High | High | Predictors were measured for the stated playing-time analysis, but the very small sample and unstable cross-validation create high analysis ROB. Temporal mismatch is retained only as applicability. | Source report, pp. 1-2 and 5-7; Methods and Table 3 |
| Liu and Hong (2026) | Boundary | Not assessed | Not assessed | Not assessed | Not assessed | Not assessed | Not formally assessed because the unit was retained as boundary evidence and was excluded from the formal PROBAST synthesis. | Source report, pp. 1-6; Sections 3.1 and 4.1-4.3 |
| Nashwan et al. (2026) | Core | Low | Low | Low | Unclear | Unclear | No domain was high, but Analysis remained unclear because reporting or safeguards were insufficient. Principal basis: Six seasons were used for training, one later season for validation and a final later season for testing. The test season was excluded from feature construction and the temporal development sequence was preserved. | Source report, pp. 1 and 3; Methods--Temporal split and Evaluation |
| Salaj (2026) | Core | Unclear | Low | Low | Unclear | Unclear | No domain was high, but Participants, Analysis remained unclear because reporting or safeguards were insufficient. Principal basis: The most recent 20% of chronologically ordered observations formed a held-out temporal test set. Target-game data were excluded and hyperparameter tuning was restricted to the training subset. | Source report, pp. 1 and 3-6; Modeling Approach and Results |
| Simonelli et al. (2026) — next-day fatigue | Core | Low | Unclear | Unclear | High | High | High overall ROB because the Analysis domain was high. Principal basis: Within-player min-max scaling and player-specific percentile class thresholds were defined from each player's full monitoring period before the player-level split, allowing future observations to influence preprocessing and outcome definition. A 70/30 player-level holdout prevents the same player appearing in both sets and RFECV was confined to training, but no chronological holdout was used and the preprocessing leakage remains. Across-team accuracy fell to 61-71%. | Source report, pp. 2-7 and 13-14; Abstract, Data collection, Preprocessing, Machine learning approach and Limitations |
| Simonelli et al. (2026) — match-day fatigue | Core | Low | Unclear | Unclear | High | High | High overall ROB because the Analysis domain was high. Principal basis: The main random split can place observations from the same players and seasons in both sets, and player-wise normalization plus percentile class thresholds were derived from the full monitoring period before splitting. A random stratified 70/30 split and training-set RFECV were reported, with secondary cross-team transfer analyses, but no prospectively locked chronological or player-held-out primary test was used. | Source report, pp. 2-5 and 8-9; Abstract, Data collection, Machine learning approach and Limitations |
| Felice (2026) | Core | Unclear | Low | Low | Unclear | Unclear | No domain was high, but Participants, Analysis remained unclear because reporting or safeguards were insufficient. Principal basis: The next 10 sessions were predicted within one team dataset, without an independently collected team or season. The exact temporal split, preprocessing scope and hyperparameter-selection boundaries were not fully documented. | Source report, pp. 2-4; Wearable sensors, Proposed architecture and Predictive performance |
| Sheridan et al. (2024) | Core | Low | Unclear | Low | High | High | High overall ROB because the Analysis domain was high. Principal basis: Normalization and class balancing were applied before or without a clearly training-contained pipeline, and repeated player records could cross the split. A random 80/20 split was used without player-grouped or temporal testing. | Source report, pp. 1-5; Experimental design, Data collection and Statistical analysis |
| Teixeira (2024) — recovery-state classification | Comparator | Low | High | Low | High | High | Post-training RPE/session information was used in relation to a recovery state assessed before or around training, creating target–predictor temporal incoherence within the original model. Repeated athlete/session observations were also not grouped. | Source report, pp. 1-6; Procedures, RPE/TQR variables and Model evaluation |
| Mandorino et al. (2023) | Core | Low | Unclear | Low | Unclear | Unclear | No domain was high, but Predictors, Analysis remained unclear because reporting or safeguards were insufficient. Principal basis: Forward updating was combined with internal feature-selection and cross-validation stages, without player-grouped or external evaluation. The expanding history is temporally appropriate, but complete nesting of preprocessing, feature selection and tuning was not fully documented. | Source report, pp. 1-4; Data analysis and Model evaluation |
| Rossi et al. (2022) | Core | Low | Low | Low | High | High | High overall ROB because the Analysis domain was high. Principal basis: The rolling evaluation is temporally ordered, but per-player normalization appears global and the supplementary random cross-validation mixes repeated player-session observations. An expanding week-ahead evaluation repeatedly trained through week i and tested week i+1 with RFECV inside each training set. | Source report, pp. 1 and 3-5; Dataset creation, Cross-validation and Real scenario |
| Marynowicz et al. (2022) | Core | Low | Unclear | Low | High | High | High overall ROB because the Analysis domain was high. Principal basis: The same observations appear to support model fitting and reported RMSE, with no independent resampling framework for predictive error. No explicit held-out split, cross-validation, temporal test, or external validation of RMSE was reported. | Source report, pp. 1-3; Abstract, Methodology and Statistical analysis |
| Kim et al. (2022) | Core | Low | Unclear | Low | High | High | High overall ROB because the Analysis domain was high. Principal basis: Shuffling permits repeated observations from the same players across folds and grid-search nesting was not established. Stratified ten-fold cross-validation was reported, but it was neither player-grouped, session-grouped nor temporally separated. | Source report, pp. 1, 3-5; Data acquisition, Feature engineering and Model validation |
| Perri et al. (2021) | Core | Low | High | Low | High | High | High overall ROB because the Predictors, Analysis domains were high. Principal basis: Overlapping multi-day windows and ungrouped stratified folds can place closely related player-days in training and test sets. The reported three-fold procedure also states 90/10 splitting and provides no chronological or player-grouped holdout. | Source report, pp. 1-3; Dataset building, Experiment and Practical application |
| Op de Beéck et al. (2019) | Core | Low | Low | Low | High | High | High overall ROB because the Analysis domain was high. Principal basis: Chronology was respected and imputation used earlier observations, but exclusions were non-random and 35 model specifications were compared without an independent selection layer. Each player's first 80% of observations formed the learning set and the remaining 20% formed a chronological test set. | Source report, pp. 4, 7 and 9-11; Abstract, Subjects and Data analysis |
| Jaspers et al. (2018) | Core | Low | Low | Low | Unclear | Unclear | No domain was high, but Analysis remained unclear because reporting or safeguards were insufficient. Principal basis: Season 1 group data were used to test season 2, including unseen players; within-season analyses also used chronological 75/25 splits. Chronological partitions were used and no direct target-derived predictor was identified for the post-session boundary. | Source report, pp. 1-4; Abstract, Methodology and Data analysis |
| Carey et al. (2016) | Core | Low | Low | Low | High | High | High overall ROB because the Analysis domain was high. Principal basis: Training-only scaling and nested tuning were used, but repeated sessions from the same players could occur in both outer-fold training and test data. Ten-fold outer and five-fold inner nested cross-validation estimated internal performance, without player-grouped or temporal testing. | Source report, pp. 1, 3 and 5-6; Data collection, Pre-processing and Model evaluation |
| Dijkhuis et al. (2021) | Comparator | Low | High | Low | High | High | Whole-season personal baselines and labels were constructed before a random split, producing information leakage intrinsic to the original study, not merely a mismatch with this review’s intended decision point. | Source report, pp. 1, 4-7 and 13; Machine Learning and Discussion |
| Alves and Barbosa (2025) | Core | Low | Unclear | Low | High | High | High overall ROB because the Analysis domain was high. Principal basis: Normalization and target-correlated feature selection were conducted before the outer season split or were not shown to be refit within each training window, allowing future-season information into development. Expanding-window season tests preserve outcome chronology, but the preprocessing and selection pipeline is not fully nested and no independent data source was used. | Source report, pp. 10 and 13-16; Feature engineering, Normalization, Feature selection and Data splitting |
| Wong et al. (2025) | Core | Low | Unclear | Low | Unclear | Unclear | No domain was high, but Predictors, Analysis remained unclear because reporting or safeguards were insufficient. Principal basis: The 2019-2021 seasons trained the models and the later 2022 season served as a held-out test set. Numerical standardization and exploratory feature engineering were performed before splitting or without explicit training-only fitting, and model-selection nesting was not fully documented. | Source report, pp. 1 and 3-6; Feature engineering, Training and testing data, and Results |
| Shah and Zaman (2025) | Comparator | Low | Low | Low | Unclear | Unclear | The study used a prespecified stratified 80/20 split on 458 matches. Predictor timing is post-event for this review but not intrinsically biased for the reported retrospective task. Lack of independent/temporal validation and limited pipeline detail justify unclear, not automatically high, analysis ROB. | Source report, pp. 2 and 5-6; Abstract, Data collection, Preprocessing and Model evaluation |
| Tyagi et al. (2024) | Core | Low | Unclear | Low | High | High | High overall ROB because the Analysis domain was high. Principal basis: The report does not show match-grouped splitting, so multiple rows from the same match may occur in training and test data; train-only containment of preprocessing and feature selection is also unclear. A train-test evaluation was reported, but the split proportion, grouping, chronological ordering and external independence were not established. | Source report, pp. 1-2 and 5; Abstract, Methodology, Data Set Used and Results |
| Berrar et al. (2024) — exact score | Core | Low | Low | Low | Unclear | Unclear | No domain was high, but Analysis remained unclear because reporting or safeguards were insufficient. Principal basis: The final evaluation used 714 genuinely future fixtures outside the 302,691-match historical training set. The target fixtures occurred after model submission, preventing access to target outcomes during development and prediction. | Source report, pp. 1-2 and 6-7; Abstract, 2023 Soccer Prediction Challenge and Data-driven framework |
| AlMulla et al. (2023) — cumulative in-game | Core | Low | Low | Low | High | High | High overall ROB because the Analysis domain was high. Principal basis: The temporal cutoff is valid, but paired team records from the same match and repeated teams or seasons may cross folds because grouping was not described. Nested five-by-five cross-validation was used, but no match-grouped, season-held-out, chronological or external evaluation was reported. | Source report, pp. 1, 9-10 and 14-15; Abstract, Model architecture, Nested cross-validation and Partial match analysis |
| Aarons et al. (2023) | Core | Low | Low | Low | High | High | High overall ROB because the Analysis domain was high. Principal basis: The training, calibration and test partitions were independent, but random splitting may place repeated teams and seasons across partitions and does not test future-season transportability. A dedicated 20% holdout test and a separate 10% calibration set were used after ten-fold training CV, but the partitions were random rather than temporal or grouped. | Source report, pp. 1 and 4-6; Abstract, Data-mining process, Model calibration and Model evaluation |
| Choi et al. (2023) | Core | Low | Unclear | Low | High | High | High overall ROB because the Analysis domain was high. Principal basis: Previous-match construction is temporally meaningful, but the report does not fully establish train-only fitting of feature selection, sampling and model-selection steps. Internal sampling comparisons were reported, while the 2022/2023 betting simulation was not shown to be a fully frozen independent temporal evaluation of the complete pipeline. | Source report, pp. 1 and 3-7; Abstract, Data preparation, Experiments and Betting simulation |
| Głowania et al. (2023) | Core | Low | Unclear | Low | Unclear | Unclear | No domain was high, but Predictors, Analysis remained unclear because reporting or safeguards were insufficient. Principal basis: Training ended at round 17 of 2021/2022 and testing used later rounds 18 through season end, providing a chronological internal holdout. ScoreHalf, ScoreFull and bookmaker odds were excluded from the evaluated feature sets, but possible round-alignment leakage remains. | Source report, pp. 7 and 9-11; Data preparation and Experiment design |
| Filiz (2023) | Comparator | Low | Low | Low | High | High | Predictors are acceptable for the original completed-match target. Global feature selection before cross-validation creates high analysis ROB. | Source report, pp. 1-6; Abstract, Methods, Table 3 and Model evaluation |
| Yeung et al. (2023) | Core | Low | Low | Low | High | High | High overall ROB because the Analysis domain was high. Principal basis: Historical match statistics train an intermediate prediction stage, and full nesting of this two-stage pipeline within each training split was not demonstrated. The main report does not establish a temporal, season-held-out, external or fully nested evaluation of the complete forecasting pipeline. | Source report, pp. 3-8 and 10-11; Dataset, Proposed method, Performance evaluation and Discussion |
| Kapadia et al. (2019) — home-ground | Core | Low | Unclear | Low | Unclear | Unclear | No domain was high, but Predictors, Analysis remained unclear because reporting or safeguards were insufficient. Principal basis: Stratified ten-fold cross-validation was used without chronological season holdout, match grouping or external evaluation. Random folds may mix matches from the same teams and seasons, and the feature-selection procedures were not clearly nested within each training fold. | Source report, pp. 1 and 3-8; Abstract, Methodology, Data and Results |
| Hill (2022) | Core | Low | Low | Low | High | High | The same leave-one-season-out resampling framework was used for grid-search hyperparameter selection and for the reported model-performance comparisons, without a separate outer or post-selection final assessment. This selection–evaluation dependence creates high Analysis risk of bias despite season-level holdout folds and reported calibration analyses. | Source report, pp. 2 and 8-15; Abstract, Data, Methodology and Model results |
| Hsu (2021) | Core | Low | Low | Low | Unclear | Unclear | No domain was high, but Analysis remained unclear because reporting or safeguards were insufficient. Principal basis: The final season was used as an out-of-sample temporal test after model development on the preceding 31 seasons; this is internal temporal validation rather than external validation. The final-season test is temporally separated, but the development split is random or scattered and the report does not clearly establish cross-fitted first-stage CNN outputs for second-stage training. | Source report, pp. 1, 3, 6-8 and 17-19; Abstract, Methodology and Results |
| Chen et al. (2021) | Core | Low | Unclear | Low | High | High | High overall ROB because the Analysis domain was high. Principal basis: Paired home and away observations from the same physical game were not shown to be grouped, and normalization, feature selection, lag selection and model comparison were not clearly nested within folds. Only non-temporal ten-fold cross-validation was reported, with no season-held-out or chronological test of the selected pipeline. | Source report, pp. 1-2 and 5-12; Abstract, Proposed scheme and Results |
| Ballı and Özdemir (2021) | Comparator | Low | Low | Low | High | High | Predictor measurement is acceptable for the retrospective target. Multiple train/test proportions and selection of the best-performing configuration on evaluation data create high analysis ROB. | Source report, pp. 1 and 5-11; Abstract, Dataset, Models and Results |
| Horvat et al. (2020) | Core | Low | Unclear | Low | High | High | High overall ROB because the Analysis domain was high. Principal basis: Five-fold cross-validation explicitly mixes future events, and the chronological Train&Test pipeline trains on actual single-game box scores but deploys on historical team averages. Chronological disjoint and sequential tests were reported alongside temporally invalid cross-validation, but the training-deployment feature-definition mismatch undermines interpretation. | Source report, pp. 7-13; Data preparation, Prediction results and Conclusions |
| Stübinger et al. (2020) | Core | Low | Low | Low | Unclear | Unclear | No domain was high, but Analysis remained unclear because reporting or safeguards were insufficient. Principal basis: The first five matchdays formed a temporally prior training period and later matchdays were evaluated consecutively as a chronological internal test. The formation and trading periods reduce direct look-ahead, but the report does not fully establish train-only preprocessing, tuning and final pipeline locking throughout the trading period. | Source report, pp. 4 and 7-10; Data sources, Formation period and Trading period |
| Beal et al. (2020) | Core | Low | Low | Low | High | High | High overall ROB because the Analysis domain was high. Principal basis: Random resampling mixes seasons and GridSearch was reported without clear nested evaluation, although target-game statistics were not used. A random 70/30 split with ten-fold cross-validation was reported, with no chronological or future-season holdout. | Source report, pp. 1, 5 and 8-11; Abstract, Feature set and Experiments |
| Thabtah et al. (2019) | Comparator | Low | Low | High | High | High | The main internal-validity problem is global preprocessing/feature selection and outcome handling before cross-validation, not post-event predictor availability alone. | Source report, pp. 6-9; Data capture, Attribute description and Experimental analysis |
| Fahey-Gilmour et al. (2019) | Core | Low | Low | Low | High | High | High overall ROB because the Analysis domain was high. Principal basis: Physical-game records were grouped and tuning occurred within training cross-validation, but multiple pipelines were compared on the same final 2018 holdout without a second confirmatory test. Five-repeated grouped ten-fold cross-validation was used within 2013-2017, followed by a complete held-out 2018 season. | Source report, pp. 1 and 3-11; Abstract, Data collection, Statistical analysis and Model validation |
| Constantinou (2019) | Core | Low | Low | Low | Unclear | Unclear | No domain was high, but Analysis remained unclear because reporting or safeguards were insufficient. Principal basis: A predefined future competition test covered 206 matches from 26 leagues after multinational model training; this is temporal internal validation rather than external validation. The predefined test matches occurred after the training cutoff and no target-match result entered prediction. | Source report, pp. 1, 3-5 and 16-21; Abstract, Data engineering and Evaluation |
| Esme (2018) — Turkish Süper Lig pre-game outcome and risk-based prediction | Core | Low | Low | Low | High | High | High overall ROB because the Analysis domain was high. Principal basis: The half-season cutoff prevents direct target-result leakage, but k values from 1 to 20 and several abstention thresholds were compared on the same second-half evaluation set. The first 153 matches of 2015/2016 formed training data and the later 153 matches formed a chronological test set, but the final test also supported model and threshold selection and no confirmatory holdout remained. | Source report, pp. 2-5; Materials and Methods, Dataset and Experimental Results |
| Castellano (2026) — Spanish professional football post-event locomotor classification | Comparator | Low | Low | Low | High | High | Completed-match locomotor variables are valid measurements for the original descriptive classification. Global standardisation and random splitting of paired team-match records create high analysis ROB. | Source report, pp. 3-5; Participants, Predictors and Predicted Variables, Procedures and Data Analysis |
| Dong (2026) — CBA score-context post-event KPI classification | Comparator | Low | Low | Low | High | High | The score-context predictors are acceptable for the original post-event classification. Statistical and dual-model feature screening occurred before the final random split/cross-validation, retaining high analysis ROB. | Source report, pp. 1 and 3-6; Abstract, Study design, Sample and variables, Statistical analysis and Results |
| Kato et al. (2026) | Comparator | Low | Low | Low | Low | Low | Training used the 2019/2021/2023 championships, tuning was confined to the training championships, and the 2025 championship was held out for final testing. The post-event estimand remains highly inapplicable to pre-decision use, but it does not itself create internal ROB. | Source report, pp. 2 and 5-9; Abstract, Data sources, Model development and Results |
| Karakoç et al. (2026) | Comparator | Low | Low | Low | High | High | Predictors are acceptable for the original retrospective task. VIF/Boruta selection before the random split or without a clearly nested outer evaluation supports high analysis ROB. | Source report, pp. 1 and 3-6; Abstract, Data sample, Data analysis and Results |
| Luo (2025) — FIFA World Cup post-event team-match classification | Comparator | Low | Low | Low | High | High | Predictors are acceptable for post-event classification, but PCA/preprocessing before random allocation and paired team-match records create high analysis ROB. | Source report, pp. 1-4; Abstract, Data source, Principal component analysis and Neural-network modelling |
| Olaizola (2025) — Liga F post-goal in-game outcome prediction | Core | Low | Unclear | Low | High | High | High overall ROB because the Analysis domain was high. Principal basis: Leave-one-out evaluation was conducted at the goal-transition-vector level, so multiple vectors from the same match could appear in both development and validation, and RFECV or PCA was not clearly nested in an outer grouped evaluation. The 193 vectors from 58 matches were evaluated without match-grouped or season-held-out validation and several classifier and feature-reduction combinations were compared on the same data. | Source report, pp. 1-5; Abstract, Participants, Feature extraction, Feature selection and Model evaluation |
| Lima et al. (2025) | Comparator | Low | Low | Low | High | High | Measured load variables are acceptable for the reported retrospective association/classification. The small sample and ungrouped observation-level split support high analysis ROB. | Source report, pp. 2-7 and 13-15; Abstract, Procedures, Data treatment, Model development and Results |
| Young (2019) — AFL completed-match win-loss classification | Comparator | Low | Low | Low | High | High | Chronological season holdout is a strength, but random-forest depth was selected after comparing test-set performance, so the test set was reused for model selection. | Source report, pp. 1 and 3-5; Abstract, Data preparation, Feature selection and Modelling |
| Martins (2017) — completed-match pairwise football outcome classification | Comparator | Low | Low | Low | High | High | High overall ROB because the Analysis domain was high. Principal basis: the source report explicitly states that relevant features were selected from the training and testing set, so feature selection was not protected from evaluation data. The report also used 10-fold cross-validation and a four-round training/two-round testing sliding window, but these evaluation procedures do not remove bias introduced when test information contributes to feature selection. | Source report, pp. 1-2 and 8-13; Abstract, Dataset, Feature selection and Experimental validation |
| Bae (2026) — completed-season KBO playoff-advancement classification | Comparator | Low | Low | Low | High | High | Completed-season predictors fit the original target, but only 100 team-seasons were assessed with random folds and repeated teams across seasons, without a team- or future-season holdout. | Source report, pp. 1, 3-4 and 6-7; Abstract, Dataset construction, Outcome definition and Model evaluation |
| Simonelli et al. (2025) — current recovery | Core | Low | Low | Unclear | Unclear | Unclear | No domain was high, but Outcome, Analysis remained unclear because reporting or safeguards were insufficient. Principal basis: A 70/30 player-level holdout ensured no player appeared in both training and test or RFECV. Four clubs were represented, although no chronological or external-club test was reported. Player-level separation reduces repeated-measure leakage and feature selection/tuning were conducted in training, but fatigue and soreness are co-measured near-proxies for the TQR target. | Source report, pp. 2-5; Abstract, Data recording, Machine learning approach and Results |
| Teixeira (2025) — youth soccer same-session fatigue/RPE-related classification | Boundary | Not assessed | Not assessed | Not assessed | Not assessed | Not assessed | Not formally assessed because the unit was retained as boundary evidence and was excluded from the formal PROBAST synthesis. | Source report, pp. 1 and 3-11; Abstract, Study design, Variables, Preprocessing, Train-test split and Results |
| Sharma (2025) — next-week RSImod readiness classification | Core | Low | Unclear | Unclear | High | High | High overall ROB because the Analysis domain was high. Principal basis: MICE imputation, correlation-based feature selection, readiness quartiles, smallest-worthwhile-change augmentation and SMOTE-ENN were not demonstrated as fully training-fold nested. The approximately 70/30 random split mixed repeated athlete-week observations from only 17 athletes and provided no athlete-held-out or chronological future-period test. | Source report, pp. 1 and 3-5 and 9-11; Abstract, Dataset, Methodology, Data preparation and Results |
| Epp-Stobbe (2025) — post-match rugby-sevens sRPE imputation | Boundary | Not assessed | Not assessed | Not assessed | Not assessed | Not assessed | Not formally assessed because the unit was retained as boundary evidence and was excluded from the formal PROBAST synthesis. | Source report, pp. 1 and 4-5 and 10-13; Abstract, General methods, Imputation of sRPE and Discussion |
| Ren (2025) — pre-match rugby-union player KPI regression | Core | Low | Low | Low | High | High | High overall ROB because the Analysis domain was high. Principal basis: Training-set min-max scaling and PCA are shown, but repeated player-match records from one club were not separated by player, physical match or season, allowing dependence across partitions. An 80/20 internal test with training-set preprocessing and ten-fold tuning was reported, but no athlete-held-out, match-grouped, season-forward or external-club validation was performed. | Source report, pp. 1 and 3-6; Abstract, Experimental approach, Data preprocessing, Flowchart and Model evaluation |
| Baadi (2025) — European soccer in-game substitution-impact classification | Core | Low | Unclear | Low | High | High | High overall ROB because the Analysis domain was high. Principal basis: Multiple substitutions from one physical match can cross the random split, player and team identifiers can be memorized, and imputation, scaling, feature processing and class balancing were not shown to be training-only. A single random 80/20 substitution-event split was used without physical-match, club, player, season or chronological holdout validation. | Source report, pp. 1 and 3-6; Abstract, Dataset, Preprocessing, Target feature and Model evaluation |
| Ali (2023) — heterogeneous causal effect of risky substitutions | Boundary | Not assessed | Not assessed | Not assessed | Not assessed | Not assessed | Not formally assessed because the unit was retained as boundary evidence and was excluded from the formal PROBAST synthesis. | Source report, pp. 1-5 and 8-12; Abstract, Introduction, Data, Causal Forest methodology, Robustness and Limitations |
| Mohandas (2023) — European soccer in-game substitution-impact classification | Core | Low | Unclear | Low | High | High | High overall ROB because the Analysis domain was high. Principal basis: Random event-level splitting can distribute multiple substitutions from the same match across training and test sets, while identity encoding and preprocessing were not shown to be partition-protected. The study used one random 80/20 split without match-grouped, player-grouped, season-held-out or forward temporal validation. | Source report, pp. 1 and 4-7 and 20-24; Abstract, Research methodology, Data manipulation, Modelling and Validation |
| Pérez-Blanco (2026) — pre-game ordinal foul-count prediction | Core | Low | Low | Low | High | High | High overall ROB because the Analysis domain was high. Principal basis: Chronological evaluation prevents future-match leakage, but multiple discretizers and classifiers were compared over the same rolling evaluation seasons without a separate final confirmation period. The model was repeatedly trained on the two preceding seasons and tested on the following season across ten chronological test seasons. | Source report, pp. 1 and 3-11; Abstract, Proposed methodology, Sequential training table and Foul-prediction results |
| Górecki (2026) — retrospective Ekstraklasa team-strength estimation | Boundary | Not assessed | Not assessed | Not assessed | Not assessed | Not assessed | Not formally assessed because the unit was retained as boundary evidence and was excluded from the formal PROBAST synthesis. | Source report, pp. 1-3 and 6-22; Abstract, Study positioning, Data, Bayesian framework, Results and Conclusion |
| Kim et al. (2026) | Boundary | Not assessed | Not assessed | Not assessed | Not assessed | Not assessed | Not formally assessed because the unit was retained as boundary evidence and was excluded from the formal PROBAST synthesis. | Source report, pp. 18-21 and 27-29; Abstract, Data collection, Preprocessing, Applications and Limitations |
| Berrar et al. (2024) — result probabilities | Core | Low | Low | Low | Unclear | Unclear | No domain was high, but Analysis remained unclear because reporting or safeguards were insufficient. Principal basis: The final evaluation used 714 genuinely future fixtures outside the 302,691-match historical training set. The target fixtures occurred after model submission, preventing access to target outcomes during development and prediction. | Source report, pp. 1-2 and 6-7; Abstract, 2023 Soccer Prediction Challenge and Data-driven framework |
| AlMulla et al. (2023) — full-time | Comparator | Low | Low | Low | High | High | Nested five-by-five cross-validation protects hyperparameter tuning within the reported modelling framework. However, the full-time analysis used the same candidate-model evaluation pipeline as the cumulative in-game analysis, and candidate architectures were compared using reported test-set performance without a separate final confirmatory assessment. The 1,898 team-match records also represent two records per physical match, with match-level grouping not reported. These issues support High Analysis risk of bias; post-full-time timing is treated separately under applicability. | Source report, pp. 1, 9-10 and 14-15; Abstract, Model architecture, Nested cross-validation and Partial match analysis |
| Kapadia et al. (2019) — toss decision | Core | Low | Unclear | Low | Unclear | Unclear | No domain was high, but Predictors, Analysis remained unclear because reporting or safeguards were insufficient. Principal basis: Stratified ten-fold cross-validation was used without chronological season holdout, match grouping or external evaluation. Random folds may mix matches from the same teams and seasons, and the feature-selection procedures were not clearly nested within each training fold. | Source report, pp. 1 and 3-8; Abstract, Methodology, Data and Results |
| Young (2019) — AFL completed-match score-margin regression | Comparator | Low | Low | Low | High | High | Chronological season holdout is a strength, but the held-out test performance was used to choose random-forest depth; high analysis ROB therefore remains for the score-margin unit. | Source report, pp. 1 and 3-5; Abstract, Data preparation, Feature selection and Modelling |
| Sharma (2025) — next-week continuous RSImod regression | Core | Low | Unclear | Unclear | High | High | High overall ROB because the Analysis domain was high. Principal basis: MICE imputation, feature identification, quartile construction, SWC augmentation and other preprocessing were not demonstrated to be nested within the model-evaluation partitions. The class-specific regressors reused the same randomly partitioned augmented repeated-athlete data; no athlete-held-out, chronological or external test was reported. | Source report, pp. 1 and 3-5 and 9-11; Abstract, Dataset, Methodology, Data preparation and Results |
| Pérez-Blanco (2026) — pre-game draw-probability prediction | Core | Low | Low | Low | Unclear | Unclear | The first two seasons initialized the framework and subsequent seasons were evaluated chronologically using preceding information. Sequential construction limits future-match leakage, but alternative discretization and benchmark approaches were compared over the same accumulated evaluation period; the report does not clearly establish that all design choices were fixed independently of those results. Analysis risk of bias therefore remains Unclear rather than High. | Source report, pp. 1 and 3-12; Abstract, Draw-model methodology, Sequential evaluation and Draw-prediction results |

**Part B. Applicability judgements and principal rationale**

| **Study / analysis unit** | **Layer** | **Participants applicability** | **Predictors applicability** | **Outcome applicability** | Overall applicability concern | **Principal applicability rationale** | **Source page / section** |
| --- | --- | --- | --- | --- | --- | --- | --- |
| Zhong (2025) | Core | Low concern | Low concern | Low concern | Low concern | The population, prediction target, predictor timing and intended decision align with the review question. Any modelling or validation limitations are recorded under ROB rather than applicability. | Source report, pp. 6 and 12–16; dataset, pre-game feature construction, train-test evaluation and model-comparison results |
| Ouyang et al. (2024) — first two quarters / first half-related in-game model | Core | Low concern | Low concern | Low concern | Low concern | The population, prediction target, predictor timing and intended decision align with the review question. Any modelling or validation limitations are recorded under ROB rather than applicability. | Source report, pp. 4 and 7–14; algorithm, data processing, second-quarter predictor set, cross-validation and in-game results |
| Ouyang et al. (2024) — first three quarters-related in-game model | Core | Low concern | Low concern | Low concern | Low concern | The population, prediction target, predictor timing and intended decision align with the review question. Any modelling or validation limitations are recorded under ROB rather than applicability. | Source report, pp. 4 and 7–15; algorithm, data processing, third-quarter predictor set, cross-validation and in-game results |
| Ou-Yang et al. (2025) | Comparator | Low concern | High concern | Low concern | High concern | High applicability concern arises from mismatch with the review’s intended decision boundary: Complete target-game box-score differences are unavailable before or during the intended game. Internal methodological limitations are recorded separately under ROB. | Source report, pp. 4 and 7–9; dataset, model development, cross-validation and performance results |
| Yao et al. (2022) | Core | Low concern | Low concern | Low concern | Low concern | The population, prediction target, predictor timing and intended decision align with the review question. Any modelling or validation limitations are recorded under ROB rather than applicability. | Source report, pp. 7 and 9–11; Chinese Super League data, evaluation, Ranked Probability Score and calibration analyses |
| Kayhan and Watkins (2018) | Core | Low concern | Unclear concern | Low concern | Unclear concern | Applicability is unclear because alignment or reporting was insufficient for the predictors domain(s). Analysis-design limitations were not used to determine applicability. | Source report, pp. 4–5 and 10–16; dataset, snapshot construction, independent validation season, Brier scores and reliability analyses |
| Song et al. (2024) | Comparator | Low concern | High concern | Low concern | High concern | High applicability concern arises from mismatch with the review’s intended decision boundary: The complete target-match performance indicators are unavailable before the match outcome. Internal methodological limitations are recorded separately under ROB. | Source report, pp. 3 and 5–7; machine-learning methods, training-validation split, cross-validation and model-performance results |
| Osken and Onay (2022) | Core | Low concern | Unclear concern | Low concern | Unclear concern | Applicability is unclear because alignment or reporting was insufficient for the predictors domain(s). Analysis-design limitations were not used to determine applicability. | Source report, pp. 5 and 7–10; player/game data, prediction inputs, random split, held-out-season robustness check and results |
| Zhao et al. (2023) | Comparator | Low concern | High concern | Low concern | High concern | High applicability concern arises from mismatch with the review’s intended decision boundary: Node features are complete-game statistics and the graph includes links to subsequent games. Internal methodological limitations are recorded separately under ROB. | Source report, pp. 4–5 and 8–11; graph construction, dataset, feature engineering and 70/10/20 evaluation |
| Weirich et al. (2025) | Comparator | Low concern | High concern | Low concern | High concern | High applicability concern arises from mismatch with the review’s intended decision boundary: Complete-season scoring, yardage, turnover and penalty aggregates are unavailable before the season ends. Internal methodological limitations are recorded separately under ROB. | Source report, pp. 2–7; dataset, preprocessing, model architecture, random evaluation and chronological forecasting evaluation |
| Ming and Jin (2025) | Comparator | Low concern | High concern | Low concern | High concern | High applicability concern arises from mismatch with the review’s intended decision boundary: The performance indicators are not available before the set or match outcome being explained. Internal methodological limitations are recorded separately under ROB. | Source report, pp. 3, 6 and 8–12; sample, standardisation, data splitting, cross-validation, test results and external-validation description |
| Macrì Demartino et al. (2025) | Core | Low concern | Low concern | Low concern | Low concern | The population, prediction target, predictor timing and intended decision align with the review question. Any modelling or validation limitations are recorded under ROB rather than applicability. | Source report, pp. 10-17; Sections 4-5 |
| Zhang et al. (2025) | Boundary | Not assessed | Not assessed | Not assessed | Not assessed | Not formally assessed because the unit was retained as boundary evidence. | Source report, pp. 3-11; Methods and Results |
| Mohandas et al. (2026) | Boundary | Not assessed | Not assessed | Not assessed | Not assessed | Not formally assessed because the unit was retained as boundary evidence. | Source report, pp. 7-12 and 27-31; Sections 3.1-3.6, 4.8, and 5 |
| Raabe et al. (2023) | Boundary | Not assessed | Not assessed | Not assessed | Not assessed | Not formally assessed because the unit was retained as boundary evidence. | Source report, pp. 11-15; Sections 4.1-4.6 |
| Mandorino et al. (2024) | Core | Low concern | Low concern | Low concern | Low concern | The population, prediction target, predictor timing and intended decision align with the review question. Any modelling or validation limitations are recorded under ROB rather than applicability. | Source report, pp. 1-3; Methods: ML models, preprocessing, RFE, tuning, and cross-validation |
| Leppich et al. (2024) | Core | Low concern | Low concern | Low concern | Low concern | The population, prediction target, predictor timing and intended decision align with the review question. Any modelling or validation limitations are recorded under ROB rather than applicability. | Source report, pp. 1, 3, and 5-6; Training and experimental setup |
| Vallance et al. (2023) | Core | Low concern | Low concern | Low concern | Low concern | The population, prediction target, predictor timing and intended decision align with the review question. Any modelling or validation limitations are recorded under ROB rather than applicability. | Source report, pp. 1-3; Methods |
| Diouron et al. (2025) | Core | Low concern | Low concern | Low concern | Low concern | The population, prediction target, predictor timing and intended decision align with the review question. Any modelling or validation limitations are recorded under ROB rather than applicability. | Source report, pp. 1 and 4-8; machine-learning methods and delta-HR construction |
| Ulloa-Díaz et al. (2025) | Comparator | Low concern | High concern | High concern | High concern | High applicability concern arises from mismatch with the review’s intended decision boundary: The reported measurements were not available at a preseason or earlier decision point for forecasting the completed season. Internal methodological limitations are recorded separately under ROB. | Source report, pp. 1-2 and 5-7; Methods and Table 3 |
| Liu and Hong (2026) | Boundary | Not assessed | Not assessed | Not assessed | Not assessed | Not formally assessed because the unit was retained as boundary evidence. | Source report, pp. 1-6; Sections 3.1 and 4.1-4.3 |
| Nashwan et al. (2026) | Core | Low concern | Low concern | Low concern | Low concern | The population, prediction target, predictor timing and intended decision align with the review question. Any modelling or validation limitations are recorded under ROB rather than applicability. | Source report, pp. 1 and 3; Methods--Temporal split and Evaluation |
| Salaj (2026) | Core | Unclear concern | Low concern | Low concern | Unclear concern | Applicability is unclear because alignment or reporting was insufficient for the participants domain(s). Analysis-design limitations were not used to determine applicability. | Source report, pp. 1 and 3-6; Modeling Approach and Results |
| Simonelli et al. (2026) — next-day fatigue | Core | Low concern | Low concern | Low concern | Low concern | The population, prediction target, predictor timing and intended decision align with the review question. Any modelling or validation limitations are recorded under ROB rather than applicability. | Source report, pp. 2-7 and 13-14; Abstract, Data collection, Preprocessing, Machine learning approach and Limitations |
| Simonelli et al. (2026) — match-day fatigue | Core | Low concern | Low concern | Low concern | Low concern | The population, prediction target, predictor timing and intended decision align with the review question. Any modelling or validation limitations are recorded under ROB rather than applicability. | Source report, pp. 2-5 and 8-9; Abstract, Data collection, Machine learning approach and Limitations |
| Felice (2026) | Core | Unclear concern | Low concern | Low concern | Unclear concern | Applicability is unclear because alignment or reporting was insufficient for the participants domain(s). Analysis-design limitations were not used to determine applicability. | Source report, pp. 2-4; Wearable sensors, Proposed architecture and Predictive performance |
| Sheridan et al. (2024) | Core | Low concern | Low concern | Low concern | Low concern | The population, prediction target, predictor timing and intended decision align with the review question. Any modelling or validation limitations are recorded under ROB rather than applicability. | Source report, pp. 1-5; Experimental design, Data collection and Statistical analysis |
| Teixeira (2024) — recovery-state classification | Comparator | Unclear concern | High concern | Low concern | High concern | High applicability concern arises from mismatch with the review’s intended decision boundary: Post-training RPE and same-session wearable variables are unavailable at a genuine pre-training recovery decision. Internal methodological limitations are recorded separately under ROB. | Source report, pp. 1-6; Procedures, RPE/TQR variables and Model evaluation |
| Mandorino et al. (2023) | Core | Low concern | Low concern | Low concern | Low concern | The population, prediction target, predictor timing and intended decision align with the review question. Any modelling or validation limitations are recorded under ROB rather than applicability. | Source report, pp. 1-4; Data analysis and Model evaluation |
| Rossi et al. (2022) | Core | Low concern | Low concern | Low concern | Low concern | The population, prediction target, predictor timing and intended decision align with the review question. Any modelling or validation limitations are recorded under ROB rather than applicability. | Source report, pp. 1 and 3-5; Dataset creation, Cross-validation and Real scenario |
| Marynowicz et al. (2022) | Core | Low concern | Low concern | Low concern | Low concern | The population, prediction target, predictor timing and intended decision align with the review question. Any modelling or validation limitations are recorded under ROB rather than applicability. | Source report, pp. 1-3; Abstract, Methodology and Statistical analysis |
| Kim et al. (2022) | Core | Low concern | Low concern | Low concern | Low concern | The population, prediction target, predictor timing and intended decision align with the review question. Any modelling or validation limitations are recorded under ROB rather than applicability. | Source report, pp. 1, 3-5; Data acquisition, Feature engineering and Model validation |
| Perri et al. (2021) | Core | Unclear concern | High concern | Low concern | High concern | High applicability concern arises from mismatch with the review’s intended decision boundary: Longer-horizon models were built with observed intermediate wellness and load values, whereas practical use substitutes recursively predicted wellness and scheduled load. Internal methodological limitations are recorded separately under ROB. | Source report, pp. 1-3; Dataset building, Experiment and Practical application |
| Op de Beéck et al. (2019) | Core | Low concern | Low concern | Low concern | Low concern | The population, prediction target, predictor timing and intended decision align with the review question. Any modelling or validation limitations are recorded under ROB rather than applicability. | Source report, pp. 4, 7 and 9-11; Abstract, Subjects and Data analysis |
| Jaspers et al. (2018) | Core | Low concern | Low concern | Low concern | Low concern | The population, prediction target, predictor timing and intended decision align with the review question. Any modelling or validation limitations are recorded under ROB rather than applicability. | Source report, pp. 1-4; Abstract, Methodology and Data analysis |
| Carey et al. (2016) | Core | Low concern | Low concern | Low concern | Low concern | The population, prediction target, predictor timing and intended decision align with the review question. Any modelling or validation limitations are recorded under ROB rather than applicability. | Source report, pp. 1, 3 and 5-6; Data collection, Pre-processing and Model evaluation |
| Dijkhuis et al. (2021) | Comparator | Low concern | High concern | Low concern | High concern | High applicability concern arises from mismatch with the review’s intended decision boundary: The player-specific reference baseline uses all complete matches in the same season, including the target match and future matches. Internal methodological limitations are recorded separately under ROB. | Source report, pp. 1, 4-7 and 13; Machine Learning and Discussion |
| Alves and Barbosa (2025) | Core | Low concern | Low concern | Low concern | Low concern | The population, prediction target, predictor timing and intended decision align with the review question. Any modelling or validation limitations are recorded under ROB rather than applicability. | Source report, pp. 10 and 13-16; Feature engineering, Normalization, Feature selection and Data splitting |
| Wong et al. (2025) | Core | Low concern | Low concern | Low concern | Low concern | The population, prediction target, predictor timing and intended decision align with the review question. Any modelling or validation limitations are recorded under ROB rather than applicability. | Source report, pp. 1 and 3-6; Feature engineering, Training and testing data, and Results |
| Shah and Zaman (2025) | Comparator | Low concern | High concern | High concern | High concern | High applicability concern arises from mismatch with the review’s intended decision boundary: Second-innings score is known only as the chase unfolds or after it is completed and is directly linked to whether the chase succeeds. Internal methodological limitations are recorded separately under ROB. | Source report, pp. 2 and 5-6; Abstract, Data collection, Preprocessing and Model evaluation |
| Tyagi et al. (2024) | Core | Low concern | Low concern | Low concern | Low concern | The population, prediction target, predictor timing and intended decision align with the review question. Any modelling or validation limitations are recorded under ROB rather than applicability. | Source report, pp. 1-2 and 5; Abstract, Methodology, Data Set Used and Results |
| Berrar et al. (2024) — exact score | Core | Low concern | Low concern | Low concern | Low concern | The population, prediction target, predictor timing and intended decision align with the review question. Any modelling or validation limitations are recorded under ROB rather than applicability. | Source report, pp. 1-2 and 6-7; Abstract, 2023 Soccer Prediction Challenge and Data-driven framework |
| AlMulla et al. (2023) — cumulative in-game | Core | Low concern | Low concern | Low concern | Low concern | The population, prediction target, predictor timing and intended decision align with the review question. Any modelling or validation limitations are recorded under ROB rather than applicability. | Source report, pp. 1, 9-10 and 14-15; Abstract, Model architecture, Nested cross-validation and Partial match analysis |
| Aarons et al. (2023) | Core | Low concern | Low concern | Low concern | Low concern | The population, prediction target, predictor timing and intended decision align with the review question. Any modelling or validation limitations are recorded under ROB rather than applicability. | Source report, pp. 1 and 4-6; Abstract, Data-mining process, Model calibration and Model evaluation |
| Choi et al. (2023) | Core | Low concern | Low concern | Low concern | Low concern | The population, prediction target, predictor timing and intended decision align with the review question. Any modelling or validation limitations are recorded under ROB rather than applicability. | Source report, pp. 1 and 3-7; Abstract, Data preparation, Experiments and Betting simulation |
| Głowania et al. (2023) | Core | Low concern | Unclear concern | Low concern | Unclear concern | Applicability is unclear because alignment or reporting was insufficient for the predictors domain(s). Analysis-design limitations were not used to determine applicability. | Source report, pp. 7 and 9-11; Data preparation and Experiment design |
| Filiz (2023) | Comparator | Low concern | High concern | High concern | High concern | High applicability concern arises from mismatch with the review’s intended decision boundary: Scoring first, half-time result, later-period goals, possession, shooting accuracy and other full-match indicators are unavailable pre-game. Internal methodological limitations are recorded separately under ROB. | Source report, pp. 1-6; Abstract, Methods, Table 3 and Model evaluation |
| Yeung et al. (2023) | Core | Low concern | Low concern | Low concern | Low concern | The population, prediction target, predictor timing and intended decision align with the review question. Any modelling or validation limitations are recorded under ROB rather than applicability. | Source report, pp. 3-8 and 10-11; Dataset, Proposed method, Performance evaluation and Discussion |
| Kapadia et al. (2019) — home-ground | Core | Low concern | Low concern | Low concern | Low concern | The population, prediction target, predictor timing and intended decision align with the review question. Any modelling or validation limitations are recorded under ROB rather than applicability. | Source report, pp. 1 and 3-8; Abstract, Methodology, Data and Results |
| Hill (2022) | Core | Low concern | Low concern | Low concern | Low concern | The population, prediction target, predictor timing and intended decision align with the review question. Any modelling or validation limitations are recorded under ROB rather than applicability. | Source report, pp. 2 and 8-15; Abstract, Data, Methodology and Model results |
| Hsu (2021) | Core | Low concern | Low concern | Low concern | Low concern | The population, prediction target, predictor timing and intended decision align with the review question. Any modelling or validation limitations are recorded under ROB rather than applicability. | Source report, pp. 1, 3, 6-8 and 17-19; Abstract, Methodology and Results |
| Chen et al. (2021) | Core | Low concern | Low concern | Low concern | Low concern | The population, prediction target, predictor timing and intended decision align with the review question. Any modelling or validation limitations are recorded under ROB rather than applicability. | Source report, pp. 1-2 and 5-12; Abstract, Proposed scheme and Results |
| Ballı and Özdemir (2021) | Comparator | Low concern | High concern | High concern | High concern | High applicability concern arises from mismatch with the review’s intended decision boundary: Four Factors, DefenseOffense and hybrid inputs are calculated from the completed target-game box score and are unavailable before tip-off. Internal methodological limitations are recorded separately under ROB. | Source report, pp. 1 and 5-11; Abstract, Dataset, Models and Results |
| Horvat et al. (2020) | Core | Low concern | Low concern | Low concern | Low concern | The population, prediction target, predictor timing and intended decision align with the review question. Any modelling or validation limitations are recorded under ROB rather than applicability. | Source report, pp. 7-13; Data preparation, Prediction results and Conclusions |
| Stübinger et al. (2020) | Core | Low concern | Low concern | Low concern | Low concern | The population, prediction target, predictor timing and intended decision align with the review question. Any modelling or validation limitations are recorded under ROB rather than applicability. | Source report, pp. 4 and 7-10; Data sources, Formation period and Trading period |
| Beal et al. (2020) | Core | Low concern | Low concern | Low concern | Low concern | The population, prediction target, predictor timing and intended decision align with the review question. Any modelling or validation limitations are recorded under ROB rather than applicability. | Source report, pp. 1, 5 and 8-11; Abstract, Feature set and Experiments |
| Thabtah et al. (2019) | Comparator | Low concern | High concern | High concern | High concern | High applicability concern arises from mismatch with the review’s intended decision boundary: Field goals, three-point attempts, free throws, rebounds and other complete target-game variables are unavailable before the outcome. Internal methodological limitations are recorded separately under ROB. | Source report, pp. 6-9; Data capture, Attribute description and Experimental analysis |
| Fahey-Gilmour et al. (2019) | Core | Low concern | Low concern | Low concern | Low concern | The population, prediction target, predictor timing and intended decision align with the review question. Any modelling or validation limitations are recorded under ROB rather than applicability. | Source report, pp. 1 and 3-11; Abstract, Data collection, Statistical analysis and Model validation |
| Constantinou (2019) | Core | Low concern | Low concern | Low concern | Low concern | The population, prediction target, predictor timing and intended decision align with the review question. Any modelling or validation limitations are recorded under ROB rather than applicability. | Source report, pp. 1, 3-5 and 16-21; Abstract, Data engineering and Evaluation |
| Esme (2018) — Turkish Süper Lig pre-game outcome and risk-based prediction | Core | Low concern | Low concern | Low concern | Low concern | The population, prediction target, predictor timing and intended decision align with the review question. Any modelling or validation limitations are recorded under ROB rather than applicability. | Source report, pp. 2-5; Materials and Methods, Dataset and Experimental Results |
| Castellano (2026) — Spanish professional football post-event locomotor classification | Comparator | Low concern | High concern | High concern | High concern | High applicability concern arises from mismatch with the review’s intended decision boundary: Total-distance and high-speed-running variables for in-possession, out-of-possession and stopped-play phases are only complete after the match. Internal methodological limitations are recorded separately under ROB. | Source report, pp. 3-5; Participants, Predictors and Predicted Variables, Procedures and Data Analysis |
| Dong (2026) — CBA score-context post-event KPI classification | Comparator | Low concern | High concern | High concern | High concern | High applicability concern arises from mismatch with the review’s intended decision boundary: Game-related statistics, play-type indicators and the final score-margin context are produced during or after the completed target game. Internal methodological limitations are recorded separately under ROB. | Source report, pp. 1 and 3-6; Abstract, Study design, Sample and variables, Statistical analysis and Results |
| Kato et al. (2026) | Comparator | Low concern | High concern | High concern | High concern | High applicability concern arises from mismatch with the review’s intended decision boundary: Shot success, save, technical-fault, assist, steal and block rates are generated across the completed target match. Internal methodological limitations are recorded separately under ROB. | Source report, pp. 2 and 5-9; Abstract, Data sources, Model development and Results |
| Karakoç et al. (2026) | Comparator | Low concern | High concern | High concern | High concern | High applicability concern arises from mismatch with the review’s intended decision boundary: Conversion, xG, opponent xG, shots and other technical or tactical indicators are not fully known before or during the intended match-outcome decision. Internal methodological limitations are recorded separately under ROB. | Source report, pp. 1 and 3-6; Abstract, Data sample, Data analysis and Results |
| Luo (2025) — FIFA World Cup post-event team-match classification | Comparator | Low concern | High concern | High concern | High concern | High applicability concern arises from mismatch with the review’s intended decision boundary: Inputs include complete same-match indicators such as goals scored, goals conceded, assists, shots and possession, which are unavailable before or during an earlier decision point. Internal methodological limitations are recorded separately under ROB. | Source report, pp. 1-4; Abstract, Data source, Principal component analysis and Neural-network modelling |
| Olaizola (2025) — Liga F post-goal in-game outcome prediction | Core | Low concern | Low concern | Low concern | Low concern | The population, prediction target, predictor timing and intended decision align with the review question. Any modelling or validation limitations are recorded under ROB rather than applicability. | Source report, pp. 1-5; Abstract, Participants, Feature extraction, Feature selection and Model evaluation |
| Lima et al. (2025) | Comparator | Low concern | High concern | High concern | High concern | High applicability concern arises from mismatch with the review’s intended decision boundary: Training or match duration, RPE, sRPE and jumps are generated during the activity, while wellness is recorded the following morning. Internal methodological limitations are recorded separately under ROB. | Source report, pp. 2-7 and 13-15; Abstract, Procedures, Data treatment, Model development and Results |
| Young (2019) — AFL completed-match win-loss classification | Comparator | Low concern | High concern | High concern | High concern | High applicability concern arises from mismatch with the review’s intended decision boundary: All retained absolute and relative performance indicators are derived from the completed target match and are unavailable before play. Internal methodological limitations are recorded separately under ROB. | Source report, pp. 1 and 3-5; Abstract, Data preparation, Feature selection and Modelling |
| Martins (2017) — completed-match pairwise football outcome classification | Comparator | Low concern | High concern | High concern | High concern | High applicability concern arises from mismatch with the review’s intended decision boundary: The predictor set contains complete target-match scout and event variables, including outcome-proximal events such as goals. Internal methodological limitations are recorded separately under ROB. | Source report, pp. 1-2 and 8-13; Abstract, Dataset, Feature selection and Experimental validation |
| Bae (2026) — completed-season KBO playoff-advancement classification | Comparator | Low concern | High concern | High concern | High concern | High applicability concern arises from mismatch with the review’s intended decision boundary: ERA, WHIP, FIP and the other predictors are final completed-season statistics and are unavailable for an earlier playoff-qualification decision. Internal methodological limitations are recorded separately under ROB. | Source report, pp. 1, 3-4 and 6-7; Abstract, Dataset construction, Outcome definition and Model evaluation |
| Simonelli et al. (2025) — current recovery | Core | Low concern | Low concern | Low concern | Low concern | The population, prediction target, predictor timing and intended decision align with the review question. Any modelling or validation limitations are recorded under ROB rather than applicability. | Source report, pp. 2-5; Abstract, Data recording, Machine learning approach and Results |
| Teixeira (2025) — youth soccer same-session fatigue/RPE-related classification | Boundary | Not assessed | Not assessed | Not assessed | Not assessed | Not formally assessed because the unit was retained as boundary evidence. | Source report, pp. 1 and 3-11; Abstract, Study design, Variables, Preprocessing, Train-test split and Results |
| Sharma (2025) — next-week RSImod readiness classification | Core | Low concern | Low concern | Low concern | Low concern | The population, prediction target, predictor timing and intended decision align with the review question. Any modelling or validation limitations are recorded under ROB rather than applicability. | Source report, pp. 1 and 3-5 and 9-11; Abstract, Dataset, Methodology, Data preparation and Results |
| Epp-Stobbe (2025) — post-match rugby-sevens sRPE imputation | Boundary | Not assessed | Not assessed | Not assessed | Not assessed | Not formally assessed because the unit was retained as boundary evidence. | Source report, pp. 1 and 4-5 and 10-13; Abstract, General methods, Imputation of sRPE and Discussion |
| Ren (2025) — pre-match rugby-union player KPI regression | Core | Low concern | Low concern | Low concern | Low concern | The population, prediction target, predictor timing and intended decision align with the review question. Any modelling or validation limitations are recorded under ROB rather than applicability. | Source report, pp. 1 and 3-6; Abstract, Experimental approach, Data preprocessing, Flowchart and Model evaluation |
| Baadi (2025) — European soccer in-game substitution-impact classification | Core | Low concern | Low concern | Low concern | Low concern | The population, prediction target, predictor timing and intended decision align with the review question. Any modelling or validation limitations are recorded under ROB rather than applicability. | Source report, pp. 1 and 3-6; Abstract, Dataset, Preprocessing, Target feature and Model evaluation |
| Ali (2023) — heterogeneous causal effect of risky substitutions | Boundary | Not assessed | Not assessed | Not assessed | Not assessed | Not formally assessed because the unit was retained as boundary evidence. | Source report, pp. 1-5 and 8-12; Abstract, Introduction, Data, Causal Forest methodology, Robustness and Limitations |
| Mohandas (2023) — European soccer in-game substitution-impact classification | Core | Low concern | Low concern | Low concern | Low concern | The population, prediction target, predictor timing and intended decision align with the review question. Any modelling or validation limitations are recorded under ROB rather than applicability. | Source report, pp. 1 and 4-7 and 20-24; Abstract, Research methodology, Data manipulation, Modelling and Validation |
| Pérez-Blanco (2026) — pre-game ordinal foul-count prediction | Core | Low concern | Low concern | Low concern | Low concern | The population, prediction target, predictor timing and intended decision align with the review question. Any modelling or validation limitations are recorded under ROB rather than applicability. | Source report, pp. 1 and 3-11; Abstract, Proposed methodology, Sequential training table and Foul-prediction results |
| Górecki (2026) — retrospective Ekstraklasa team-strength estimation | Boundary | Not assessed | Not assessed | Not assessed | Not assessed | Not formally assessed because the unit was retained as boundary evidence. | Source report, pp. 1-3 and 6-22; Abstract, Study positioning, Data, Bayesian framework, Results and Conclusion |
| Kim et al. (2026) | Boundary | Not assessed | Not assessed | Not assessed | Not assessed | Not formally assessed because the unit was retained as boundary evidence. | Source report, pp. 18-21 and 27-29; Abstract, Data collection, Preprocessing, Applications and Limitations |
| Berrar et al. (2024) — result probabilities | Core | Low concern | Low concern | Low concern | Low concern | The population, prediction target, predictor timing and intended decision align with the review question. Any modelling or validation limitations are recorded under ROB rather than applicability. | Source report, pp. 1-2 and 6-7; Abstract, 2023 Soccer Prediction Challenge and Data-driven framework |
| AlMulla et al. (2023) — full-time | Comparator | Low concern | High concern | High concern | High concern | High applicability concern arises from mismatch with the review’s intended decision boundary: Complete 90-minute technical and physical information is unavailable before the final outcome is known. Internal methodological limitations are recorded separately under ROB. | Source report, pp. 1, 9-10 and 14-15; Abstract, Model architecture, Nested cross-validation and Partial match analysis |
| Kapadia et al. (2019) — toss decision | Core | Low concern | Low concern | Low concern | Low concern | The population, prediction target, predictor timing and intended decision align with the review question. Any modelling or validation limitations are recorded under ROB rather than applicability. | Source report, pp. 1 and 3-8; Abstract, Methodology, Data and Results |
| Young (2019) — AFL completed-match score-margin regression | Comparator | Low concern | High concern | High concern | High concern | High applicability concern arises from mismatch with the review’s intended decision boundary: The regression inputs are complete same-match absolute and relative performance indicators unavailable before the match ends. Internal methodological limitations are recorded separately under ROB. | Source report, pp. 1 and 3-5; Abstract, Data preparation, Feature selection and Modelling |
| Sharma (2025) — next-week continuous RSImod regression | Core | Low concern | Low concern | Low concern | Low concern | The population, prediction target, predictor timing and intended decision align with the review question. Any modelling or validation limitations are recorded under ROB rather than applicability. | Source report, pp. 1 and 3-5 and 9-11; Abstract, Dataset, Methodology, Data preparation and Results |
| Pérez-Blanco (2026) — pre-game draw-probability prediction | Core | Low concern | Low concern | Low concern | Low concern | The population, prediction target, predictor timing and intended decision align with the review question. Any modelling or validation limitations are recorded under ROB rather than applicability. | Source report, pp. 1 and 3-12; Abstract, Draw-model methodology, Sequential evaluation and Draw-prediction results |
